# Protein succinylome analysis identifies citrate synthase as a central regulator of osteoclast metabolic activity

**DOI:** 10.1111/febs.70090

**Published:** 2025-04-02

**Authors:** Dayoung Yu, Yue Gao, Marcin Luzarowski, Elisabeth Seebach, Thomas Heitkamp, Michael Börsch, Thomas Ruppert, Katharina F. Kubatzky

**Affiliations:** ^1^ Medical Faculty Heidelberg, Department of Infectious Diseases, Medical Microbiology and Hygiene Heidelberg University Heidelberg Germany; ^2^ Department of Infectious Diseases University Hospital Heidelberg Germany; ^3^ Core Facility for Mass Spectrometry and Proteomics Center for Molecular Biology at Heidelberg University (ZMBH) Heidelberg Germany; ^4^ Single Molecule Microscopy Group Jena University Hospital Jena Germany

**Keywords:** citrate synthase, metabolism, mitochondria, osteoclast, post‐translational modification, PTM scan, RANKL, succinylation

## Abstract

Tumour necrosis factor ligand superfamily member 11 (TNFSF11; RANKL) and macrophage colony‐stimulating factor 1 receptor (M‐CSF) differentiate macrophages into osteoclasts. This process is characterised by changes in metabolic activity that support energy‐consuming processes. Treatment with RANKL triggers a phenotype of accelerated metabolism with enhanced glycolysis and an initial disruption of the tricarboxylic acid cycle (TCA) through increased expression of the enzyme aconitate decarboxylase (ACOD1), which results in an upregulation of intracellular succinate levels. Succinate then causes post‐translational succinylation of lysine residues. ACOD1 as an inducer of protein succinylation and the desuccinylase NAD‐dependent protein deacylase sirtuin‐5, mitochondrial (SIRT5) are regulated differentially, and the initially high expression of ACOD1 decreases towards the end of differentiation, whereas SIRT5 levels increase. To mimic the effect of protein succinylation, diethyl succinate or a SIRT5 inhibitor was added during differentiation, which reduced the formation of large osteoclasts, showing its relevance for osteoclastogenesis. To identify succinylated proteins, we used an immunoaffinity‐based liquid chromatography–tandem mass spectrometry (LC–MS/MS) approach. Most lysine succinylated proteins were mitochondrial metabolic enzymes. Citrate synthase (CS), the enzyme catalysing the first reaction of the TCA cycle, showed a notable difference in succinylation levels before and after RANKL stimulation, with succinylation detected exclusively in stimulated cells. Immunoprecipitation assays confirmed CS succinylation. Using whole cell extracts, we observed that RANKL treatment decreased CS activity in a concentration‐dependent manner. This suggests that CS could be critical in the context of energy production during osteoclastogenesis and that protein succinylation modulates the differentiation program of osteoclasts.

## Graphical abstract

RANKL treatment induces expression of the aconitate decarboxylase (ACOD1) in differentiating osteoclasts, which increases succinate levels. The initially high expression of ACOD1 decreases towards the end of differentiation when the levels of the desuccinylase SIRT5 increase. Succinylation of citrate synthase (CS) was detected exclusively in stimulated cells where it decreased CS activity in a concentration‐dependent manner. CS seems to be a critical factor for energy production during osteoclastogenesis.

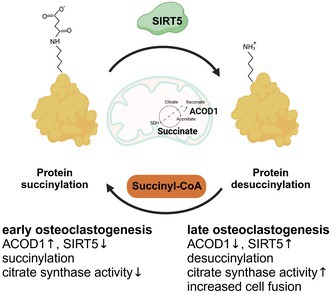

**Abbreviations**
ACLY: ATP‐citrate lyase
ACN: acetonitrile
ACOD1: aconitate decarboxylase
ATP5B: ATP F1 subunit beta, mitochondrial
CAA: chloroacetic acid
CBP: CREB binding protein
CPT1A: carnitine palmitoyl transferase 1A
CS: citrate synthase
DAMP: danger associated molecular pattern
DS: diethyl succinate
HAT: histone acetyl transferase
HIF: hypoxia inducible factor
IL: interleukin
KAT2A: lysine acetyl transferase
LC–MS/MS: liquid chromatography–tandem mass spectrometry
LDH: lactate dehydrogenase
LPS: lipopolysaccharide
M‐CSF: macrophage colony‐stimulating factor
MDH2: malate dehydrogenase 2
NFATc1: nuclear factor of activated T cells
PHD: prolyldehydrogenase
PTM: post‐translational modification
RANKL/RL: receptor of activated NF‐kB
SHMT2: serine hydroxymethyltransferase
SIRT5: silent mating type information regulation 2 homologue) 5
SUCNR: succinate receptor
TCA: tricarboxylic cycle
TCEP: tris(2‐chlorehyl)phosphate
TEAB: tetraethylammonium bromide
TFA: trifluoroacetic acid
TNF‐alpha: tumour necrosis factor alpha

## Introduction

Osteoclasts are specialised myeloid cells that can resorb bone material, a process crucial for bone remodelling and homeostasis [1]. The differentiation of osteoclasts from a monocyte precursor is regulated by two cytokines that are produced by bone‐building osteoblasts, receptor activator of nuclear factor‐kappa B (RANK) ligand and macrophage colony‐stimulating factor (M‐CSF). Under inflammatory conditions, pro‐inflammatory cytokines such as interleukin (IL)‐1β, IL‐6 and tumour necrosis factor (TNF)‐α can additionally support the differentiation process [2]. Therefore, there is a dynamic interaction between the skeletal and the immune system where immune cells contribute to bone remodelling and influence the differentiation and activity of osteoclasts. As innate immune cells, osteoclasts can also contribute to an ongoing immune reaction, but this role is less well‐described [3, 4].

During an infection, cellular metabolic activity drastically changes to adapt the function of the cell to the new stimulus. Just like rapidly dividing cells, activated immune cells depend on the availability of glucose and its quick degradation through glycolysis [5]. Although glycolysis yields fewer molecules of ATP than oxidative phosphorylation, it is able to provide energy much faster and is therefore the preferred metabolic pathway [6]. In addition to the rapid production of ATP, glycolysis‐related intermediates are critical for synthesising nucleotides, amino acids and lipids, which are essential for cell growth and the production of effector molecules. In inflamed tissues, oxygen levels are often low due to vascular damage or high metabolic demand. Glycolysis allows immune cells to function effectively in these hypoxic conditions, as it does not rely on oxygen. Glycolysis can funnel metabolites into the pentose phosphate pathway (PPP), producing NADPH. NADPH is essential for generating reactive oxygen species (ROS) used by phagocytes to kill pathogens. Accumulation of glycolytic intermediates, such as lactate, can modulate the immune environment. For instance, lactate can act as a signalling molecule to influence immune cell recruitment and polarisation [7]. Anti‐inflammatory cells, on the other hand, make use of the Krebs cycle coupled to oxidative phosphorylation to generate energy through catabolic reactions. For osteoclasts, it has been shown that both glycolysis and oxidative phosphorylation are essential for successful differentiation [2]. Increased glycolysis supports the production of biosynthetic substrates through anabolic pathways, while the abundant presence of mitochondria helps to produce large amounts of ATP that are needed for osteoclast function [8]. Initially, differentiating osteoclasts switch to accelerated glycolysis, whereas oxidative phosphorylation is especially important for the maturation and function of osteoclasts [9, 10]. A block of mitochondrial complexes, therefore, results in a retardation of the differentiation process [9].

The consumption of glucose can lead to the accumulation of metabolites such as lactate, pyruvate or succinate, and these molecules can influence cellular activity [11]. Glucose is degraded into two molecules of pyruvate. Pyruvate can then enter the TCA cycle or, for example, under hypoxic conditions, can be converted into lactate through lactate dehydrogenase (LDH). Both metabolites, pyruvate and lactate, can modulate osteoclast differentiation. The addition of low amounts of pyruvate was found to enhance osteoclast formation through an increase in acidification of the medium, ATP production and mitochondrial activity [9, 12]. Interestingly, RANKL treatment caused an upregulation of LDH, which eventually results in enhanced glycolysis, mitochondrial respiration and ATP production [13]. The effect of accumulating lactate on mitochondrial activity, however, seems to be indirect as it enhances the expression of Nuclear Factor of activated T cells 1 (NFATc1) and the expression of osteoclast‐specific genes downstream, which resulted in the observed mitochondrial upregulation [13].

In the TCA cycle, succinate is a central metabolite that affects osteoclastogenesis through three different pathways. Succinate can act as a pro‐inflammatory stimulus on macrophages and monocytes, resulting in increased IL‐1β production through stabilisation of hypoxia‐induced factor (HIF)‐1α as a consequence of substrate‐mediated inhibition of prolyl hydroxylase activity (PHD) [14]. IL‐1β is a key player in the pro‐inflammatory actions of innate immune cells but additionally has osteoclastogenic properties and supports osteoclast formation in the presence of RANKL [15, 16]. Extracellular succinate can act as a ligand for the G‐protein‐coupled receptor GPR91, also referred to as the succinate receptor SUCNR1. Succinate released from activated macrophages activates GPR91 and exacerbates rheumatoid arthritis through increased IL‐1β production [17]. The absence of GPR91 on dendritic cells (DCs) consequently decreased rheumatoid arthritis through an inhibition of DC activation and reduced expansion of Th17 cells [18]. Additionally, succinate can be added to lysine residues on proteins leading to a post‐translational modification called succinylation [19]. Especially in the mitochondria, protein succinylation seems to be a concentration‐dependent process that does not need to be facilitated by enzymes [20]. The enzymes carnitine palmitoyl transferase 1A (CPT1A), histone acetyltransferase 1 (HAT1) and lysine acetyltransferase 2A (KAT2A) and the HAT p300/CBP complex were found to act as succinylases, especially in the cytoplasm and the nucleus [21, 22, 23, 24]. Removal of succinylation is supported by the desuccinylase SIRT5, a member of the sirtuin family of NAD^+^‐dependent deacetylases [19]. Due to the comparatively high molecular weight, this modification can be expected to affect both protein structure and function [20, 25]. While a detailed understanding of protein succinylation and its role in disease is still evolving, data from animal knockout models suggest its involvement in several pathological conditions. Many studies have linked changes in protein succinylation to increased cardiovascular diseases, disruption of vascular homeostasis and endothelial cell function, thus contributing to conditions such as heart failure, atherosclerosis and various types of cancers as reviewed in [20]. Succinylation is particularly relevant in the context of mitochondrial function. Increased succinylation of mitochondrial proteins usually affects oxidative phosphorylation negatively and reduces cellular respiration, shifting metabolism towards glycolysis. So far, not much is known about the effects of protein succinylation under microbial inflammatory conditions or for auto‐immunity, although dysregulated succinate production has been associated with the modulation of immune cell function and the production of inflammatory cytokines [14]. Tannahill *et al*. describe that an increase in intracellular succinate after LPS stimulation could be attributed to enhanced glutamine‐dependent anaplerosis and resulted in an increase in cellular protein succinylation by a factor two, and their mass spectrometry analysis identified a number of metabolic proteins that are succinylated after LPS treatment. However, a functional analysis of these succinylation sites was not done, and it remains to be investigated whether protein succinylation is a prerequisite for efficient inflammation or a consequence of the changed metabolic activity of activated macrophages. The role of protein succinylation in bone‐related diseases has not been investigated so far. However, given the involvement of protein succinylation in metabolic processes and the observed shift in metabolic activity in activated immune cells and osteoclasts, the resulting changes in succinylation might be particularly important for inflammatory bone diseases. Under the auto‐inflammatory conditions of rheumatoid arthritis, increased levels of succinate can be found in the synovial fluid of patients [26]. It is possible that in addition to the known effects of GPR91 and IL‐1β, altered succinylation patterns could contribute to the progression of inflammatory bone diseases. Indeed, changes in mitochondrial function have been associated with bone‐related diseases [27, 28], and succinylation could therefore be a relevant contributing factor.

In this manuscript, we characterised the succinylome in differentiating osteoclasts and identified specific, succinylated proteins. Our data suggest that protein succinylation contributes to the metabolic reprogramming of differentiating osteoclasts after stimulation with RANKL towards glycolysis by targeting the gate‐keeping enzyme of the TCA cycle, citrate synthase.

## Results

### Succinate accumulates during osteoclastogenesis

**Fig. 1:**
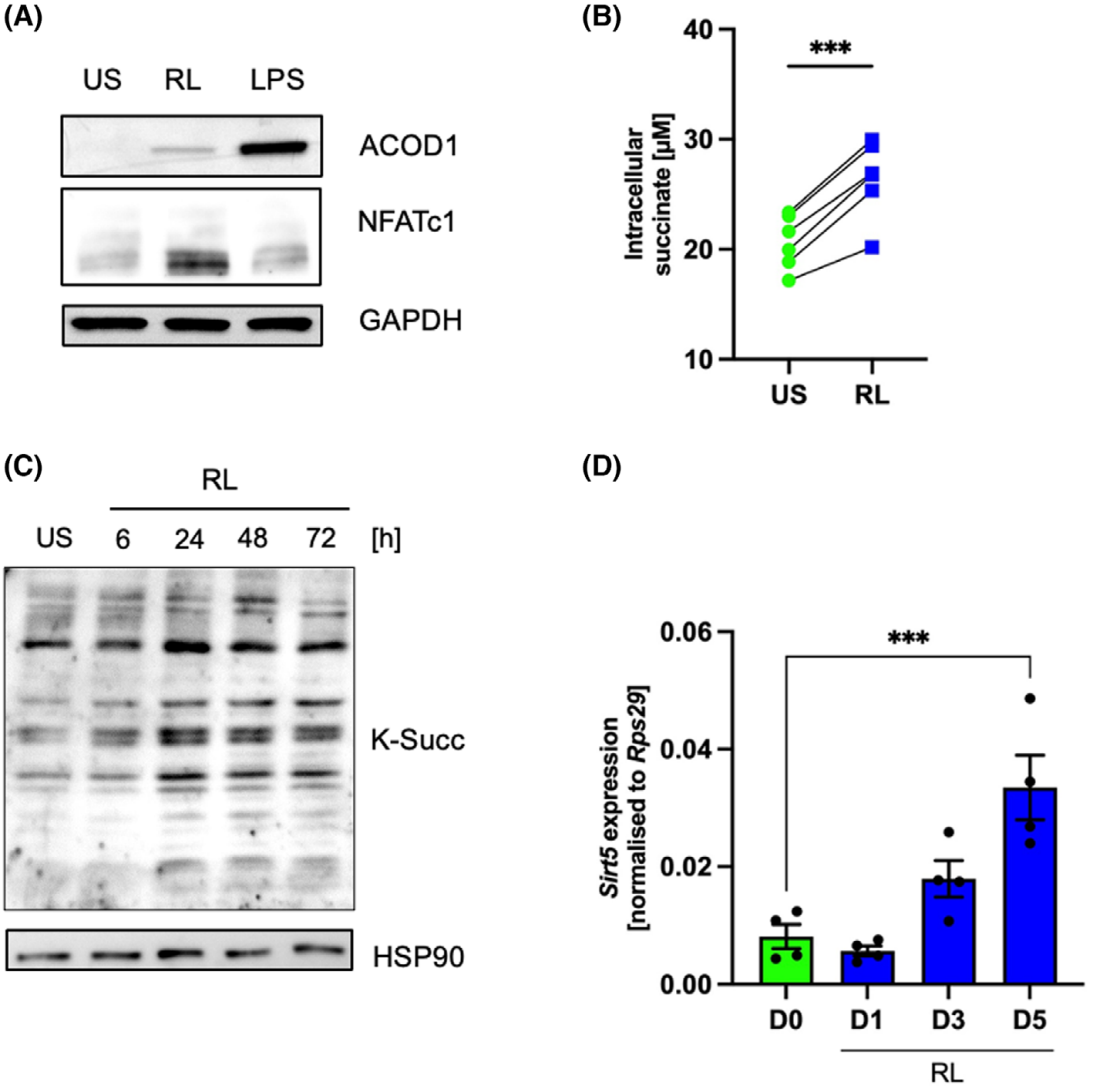
RANKL stimulation induces succinate accumulation and enhances succinylation. (A) Western blot analysis of ACOD1 and NFATc1 in cell lysates of RAW 264.7 cells treated with RANKL (RL) at 50 ng·mL^−1^ or lipopolysaccharide (LPS) at 100 ng·mL^−1^ for 24 h compared to unstimulated cells (US). GAPDH was used as a loading control (*n* = 4). (B) Intracellular succinate concentrations in RAW 264.7 cells with or without RANKL stimulation for 1 day (paired two‐tailed *t*‐test, *n* = 4, ***P < 0.001). (C) Kinetic expression of protein succinylation induced by RANKL. RAW 264.7 cells were stimulated with RANKL at a series of time points (Day 0 to Day 3). HSP90 was used as a loading control (*n* = 3). (D) Transcriptional levels of *Sirt5* expression as mean with SEM. RAW 264.7 cells were treated with RANKL at a series of time points (Day 0 to Day 5). Statistical differences between groups were calculated by ordinary one‐way ANOVA, *n* = 4, ***P < 0.001.

We had recently reported that RANKL stimulation triggers *Irg1* gene induction in RAW 264.7 cells, suggesting that the enzyme aconitate decarboxylase 1 (ACOD1) plays a role in osteoclast differentiation [29]. Here, we show that also on the protein level ACOD1 increases after treatment with 50 ng·mL^−1^ RANKL or 100 ng·mL^−1^ LPS, which was used as a positive control, after 24 h (Fig. 1A; Fig. S1A). The expression of NFATc1 is shown as a proof of successful induction of osteoclastogenesis by RANKL (lower panel, Fig. 1A; Fig. S1A). ACOD1 causes the production of itaconate from isocitrate, which then acts as an inhibitor for the enzyme succinate dehydrogenase (SDH) of the complex II of the electron transport chain. Therefore, we investigated if RANKL treatment would result in a measurable accumulation of intracellular succinate after 24 h of RANKL treatment (Fig. 1B). Indeed, we observed a statistically significant rise in intracellular succinate levels from 20 to 26 μm.

Intracellular succinate can be a source for succinylation of proteins in various cell compartments. Especially in the mitochondria, the intermediate metabolite succinyl coenzyme A is attached nonenzymatically to accessible lysine residues. Therefore, we analysed protein succinylation during osteoclast differentiation of RAW 264.7 macrophages treated for 24, 48 and 72 h with 50 ng·mL^−1^ RANKL or left untreated as a control (US). Western blot analysis using a pan‐succinylation antibody shows that protein succinylation peaks at 24 h and remains stable until 72 h in RAW 264.7 macrophages (Fig. 1C). Therefore, for subsequent experiments, we selected the 48‐h time point as it represents a well‐advanced stage of osteoclast formation. Comparable changes in protein succinylation were observed for bone marrow‐derived macrophages (Fig. S1B). While succinylation is thought to occur nonenzymatically in the mitochondria and depends on the amount of available succinate in the cell, desuccinylation is supported by the enzyme SIRT5 that removes succinyl moieties from lysine residues. We therefore investigated whether the observed increase in protein succinylation was supported by a decrease in *Sirt5* expression. Figure 1D shows that there is a slight decrease in gene expression after 24 h that remained statistically non‐significant and that *Sirt5* levels are induced at later stages and are highest on Day 5, where a significant induction compared to Day 0 can be observed.

### ACOD1 and SIRT5 are differentially regulated during osteoclastogenesis

**Fig. 2:**
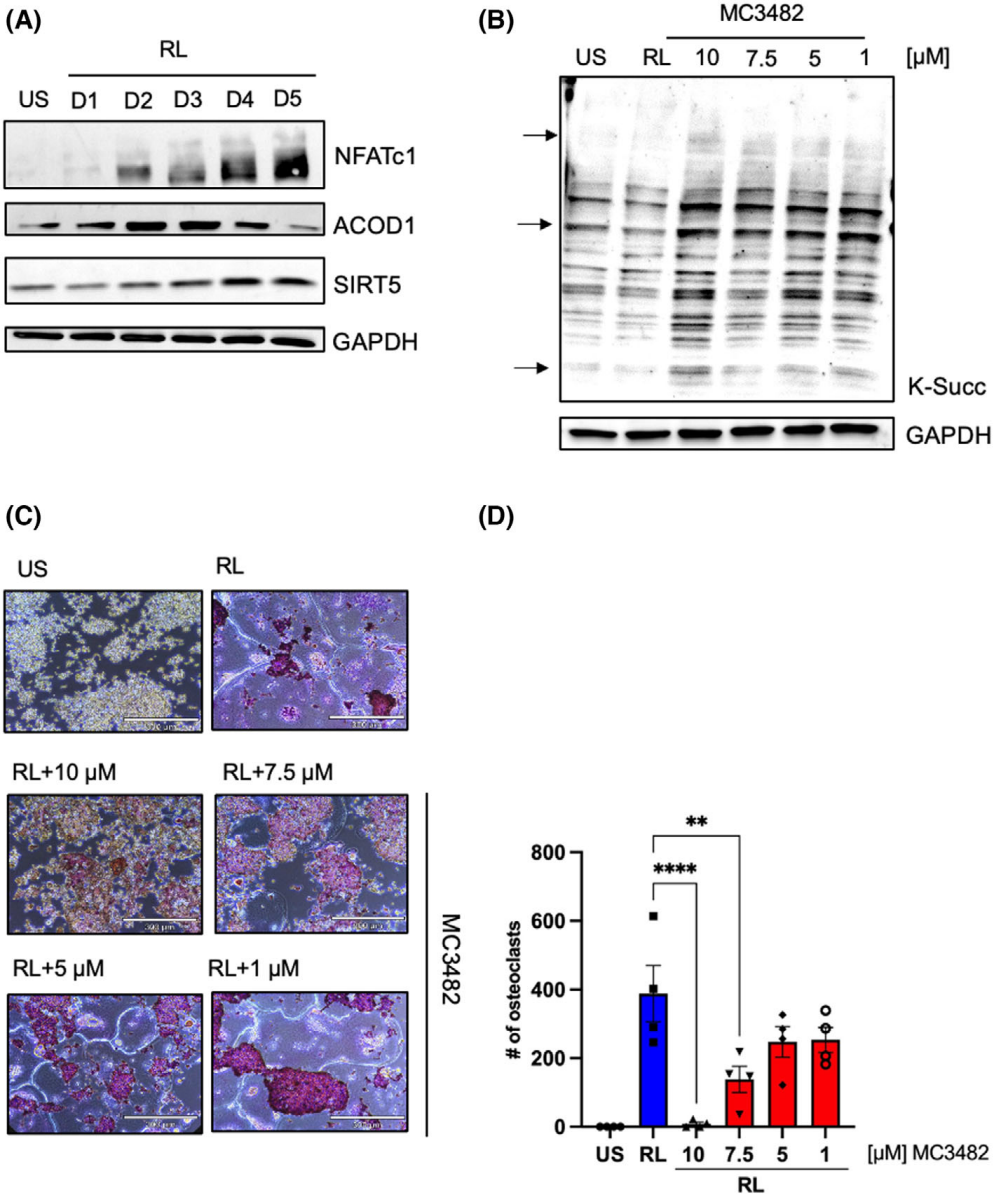
Differential expression of ACOD1 and SIRT5 during osteoclastogenesis. (A) Western blot analysis of ACOD1 and SIRT5 expression with RANKL stimulation at a series of time points (Day 0 to Day 5). NFATc1 was used as a marker for ongoing osteoclastogenesis, GAPDH was used as a loading control (*n* = 3). (B) Western blot analysis of lysine succinylation in whole cell lysates. RAW 264.7 cells were treated with RANKL (RL) or different concentrations of the SIRT5 inhibitor MC3482 for 2 days. GAPDH was used as a loading control. The arrows indicate examples of increased succinylation levels of substrates following SIRT5 inhibitor treatment (*n* = 3). (C, D) TRAP assay analysis of RANKL‐derived osteoclast formation in RAW 264.7 cells in the presence or absence of the SIRT5 inhibitor. After pre‐incubation with DMSO or different concentrations of MC3482 for an hour, RANKL (RL) was given for 5 days. Half of the culture volume was refreshed on day 3. (C) Microscopic pictures of TRAP‐positive RANKL‐dependent fused osteoclasts. An unstimulated sample was included as control. Scale bar represents 300 μm. (D) Number of counted osteoclasts as mean with SEM. Statistical differences between groups were calculated by ordinary one‐way ANOVA, *n* = 4, ****P < 0.0001, **P < 0.01.

When we compared the protein expression of ACOD1 and SIRT5 during osteoclastogenesis (Days 0–5), we saw that RANKL treatment led to an upregulation of ACOD1 until Day 3 followed by a considerable downregulation on Days 4 and 5 (Fig. 2A, Fig. S2A). SIRT5 levels, on the other hand, were low initially and expression was upregulated late (Days 4 and 5). NFATc1 was included as a control for ongoing osteoclastogenesis, while GAPDH was used to verify equal loading of samples. A comparable regulation was observed for BMDM, where ACOD1 was highly expressed on Days 1 and 2 and SIRT5 on Days 4 and 5 (Fig. S2B).

Next, we investigated whether the addition of the SIRT5 inhibitor MC3482 at nontoxic concentrations would impact osteoclast differentiation (Fig. S2C). Figure 2B shows that, like RANKL treatment, the addition of the SIRT5 inhibitor increased succinylation levels in whole cell lysates (Fig. S2D). The addition of increasing concentrations of MC3482 to differentiating osteoclasts resulted in a marked decrease in the number of osteoclasts that was concentration‐dependent (Fig. 2C,D). Interestingly, despite a statistically significant decrease in osteoclasts for 10 and 7.5 μM of inhibitor, the addition of these nontoxic concentrations of MC3482 did not cause a significant downregulation of osteoclast gene expression (Fig. S2E–I).

### Addition of diethyl succinate impacts osteoclast fusion but not differentiation

**Fig. 3:**
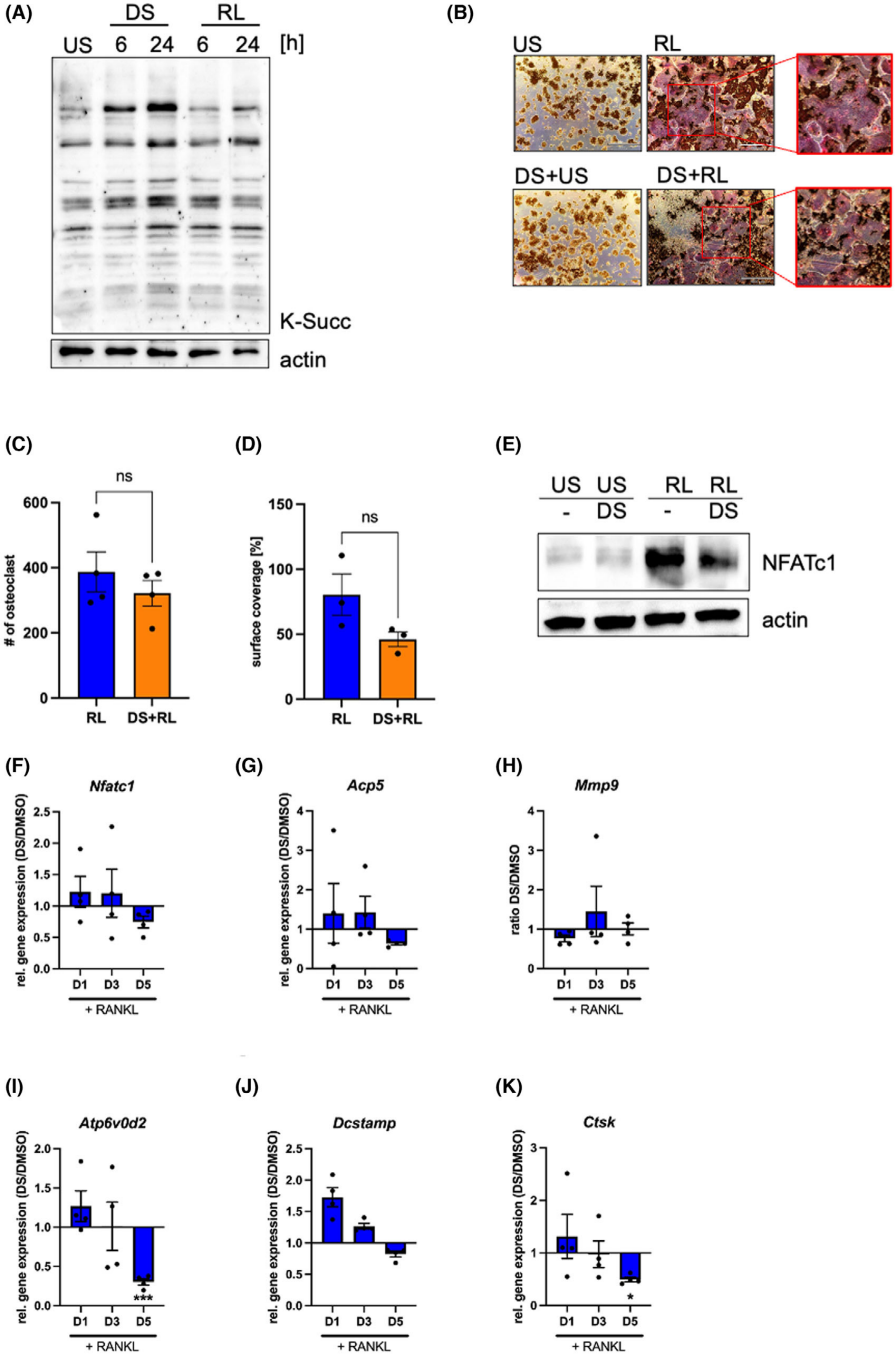
Diethyl succinate decreases osteoclast fusion but not differentiation. (A) Western blot analysis of pan‐succinylation. RAW 264.7 cells were stimulated with 5 mm diethyl succinate (DS), 50 ng·mL^−1^ RANKL (RL) for 6 and 24 h (*n* = 2). (B) TRAP analysis of RANKL‐derived osteoclast formation in the presence of cell‐permeable succinate. After pre‐incubation with DMSO or DS for an hour, RAW 264.7 cells were treated with RANKL and stained after 5 days. Scale bar represents 1000 μm. Representative pictures are shown (*n* = 4). (C) Quantification of multi‐nucleated osteoclasts observed by TRAP analysis. Cells containing more than 3 nuclei were counted as osteoclasts. Data represent biologically independent samples with line at mean and SEM (paired two‐tailed *t*‐test, *n* = 4). (D) Percentage of surface coverage of osteoclasts. The entire culture area was considered as 100% and the area occupied by osteoclasts was quantified accordingly. Data represent biologically independent samples with line at mean and SEM (paired two‐tailed *t*‐test, *n* = 3). (E) Protein level of osteoclastic transcription factor NFATc1. RAW 264.7 cells after an hour pre‐stimulation of DMSO or DS were subsequently treated with RANKL overnight (*n* = 4). (F–K) Transcriptional levels of osteoclastic fusion markers (F) *Nfatc1*, (G) *Acp5*, H) *Mmp9*, (I) *Atp6v0d2*, (J) *Dcstamp* and (K) *Ctsk*. RAW 264.7 cells were pre‐incubated with DMSO or DS, followed by RANKL stimulation, and collected on day 1, 3, 5. The data are displayed a relative gene expression between DS/RANKL and DMSO/RANKL‐treated samples. Each data point indicates biologically independent samples, the error bars indicate± SEM. Statistical differences between groups were calculated by ordinary one‐way ANOVA between treatment groups at the same day. The raw data used for the statistical analysis can be found in Fig. S3 (*n* = 4). *** <0.001, * <0.05.

To increase protein succinylation directly, we next set up a model where diethyl succinate was used to mimic the RANKL‐induced increase in intracellular succinate. RAW 264.7 macrophages were treated with 5 mm diethyl succinate for 6 and 24 h, respectively. Western blot analysis of cell lysates showed that, like RANKL treatment, diethyl succinate increased the level of protein succinylation in whole cell lysates, especially after 24 h (Fig. 3A). Next, we investigated whether this increase in protein succinylation would result in changes in osteoclast differentiation. Thus, we set up a TRAP assay using RAW 264.7 macrophages and 50 ng·mL^−1^ RANKL in the presence or absence of diethyl succinate (Fig. 3B). Analysis of TRAP‐positive, multinucleated cells showed that the presence of diethyl succinate changed the morphology of RANKL‐derived osteoclast formation in RAW 264.7 cells. While the total number of osteoclasts did not vary much between control cells and DS‐treated cells (Fig. 3C), the surface area of the obtained osteoclasts differentiated in the presence of diethyl succinate was smaller (Fig. 3D), suggesting a defect in osteoclast fusion, although this decrease was not statistically significant.

When we checked the activation of the osteoclastogenic transcription factor NFATc1 after RANKL stimulation for 24 h with or without diethyl succinate supplementation, we observed that NFATc1 was reproducibly lower expressed on the protein level in the presence of diethyl succinate (Fig. 3E; Fig. S3A). Next, we investigated whether this resulted in a significant reduction of osteoclast genes (Fig. 3F–K; Fig. S3B–G). Figure 3F–K show that despite an initial upregulation of all investigated RANKL‐mediated genes, the induction of the fusion markers *Atp6v0d2* and *Dcstamp* is reduced on Day 5 (Fig. 3I,J) [30]. This finding corroborates the data obtained in the TRAP assays (Fig. 3B), showing that osteoclast differentiation is induced to a similar extent in the presence and absence of diethyl succinate. In addition to *Atp6v0d2* and *Dcstamp*, the expression of the protease Cathepsin K (*Ctsk*) is also impaired by diethyl succinate treatment, but statistical significance is only seen for *Atp6v0d2* and *Ctsk* on Day 5. *Nfatc1* and *Mmp9* remained unaffected on Days 1 and 3 but show a reduction on Day 5 that remains statistically unsignificant (Fig. 3F–H) [31, 32, 33]. Due to the decreased expression of genes needed for the fusion of osteoclasts at late time points, further fusion does not take place, ultimately resulting in reduced osteoclast size (Fig. 3I,J). These data could also be corroborated for BMDM, where DS treatment resulted in a reduction in large osteoclasts (Fig. S3H,I) that was caused by a significant decrease in *Dcstamp* expression (Fig. S3J–N).

### Analysis of protein succinylation in RAW 264.7 cells stimulated by RANKL

To analyse lysine succinylation during osteoclastogenesis, we employed PTMScan®, an immunoaffinity‐based quantitative proteomics method. Proteins were first digested with trypsin, then peptides were labelled using dimethyl labelling, and finally succinylated peptides were enriched using specific antibodies (Fig. S4A). The succinylated peptides were subsequently identified and quantified using liquid chromatography–tandem mass spectrometry (LC–MS/MS). We conducted this analysis on six biological replicates from three independent experiments with RAW 264.7 cells treated with RANKL for 48 h or left untreated (US). Western blot controls are shown in Fig. S4B.

**Fig. 4:**
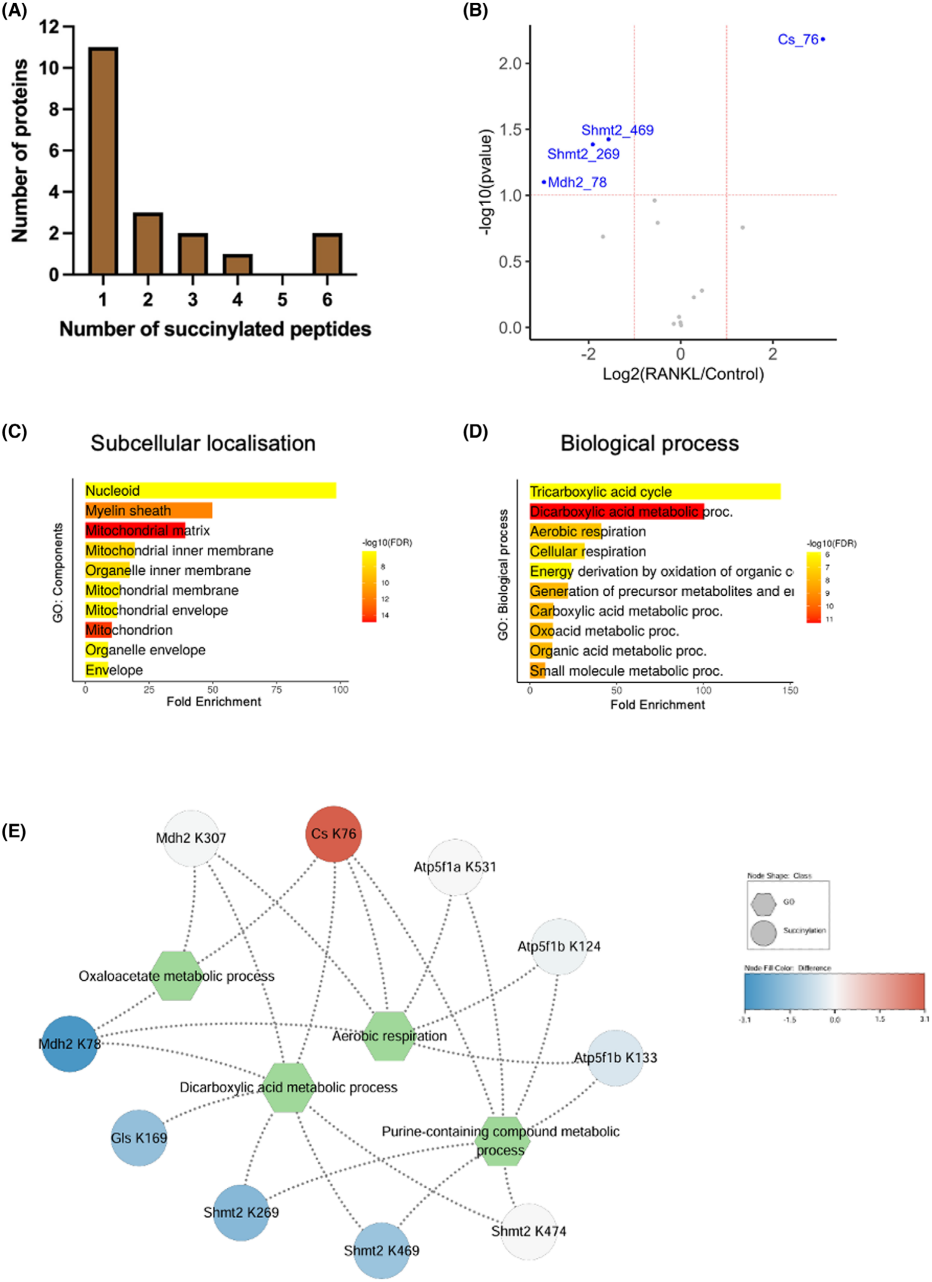
Quantitative proteomic analysis of succinylation during osteoclastogenesis. (A) shows the number of succinylation sites per individual protein. The quantitative data obtained after PTMscan analysis can be found in (Table S1) (*n* = 3). (B) Volcano plot of the RANKL‐induced protein succinylation obtained from 3 biologically independent experiments representing –log_10_(*P*‐value) on y‐axis and log_2_(fold change) on x‐axis. Significantly upregulated succinylation sites were depicted with threshold of ‐log_10_(*P*‐value) ≥1, and the ratio of log_2_(fold change) ≥1. (C, D) GO‐term enrichment analysis of succinylated proteins (*n* = 3). The bar chart shows the enriched Gene Ontology (GO) terms identified in the (C) cellular component and (D) biological process category. Displayed are 10 of the most enriched GO terms with the False Discovery Rate (FDR) threshold set to 0.05 as described in the methods section. (E) A representative network of GO biological process and succinylation level. Using STRING web database, GO biological process was analysed with a medium confidence score of ≥0.4. The representative network visualises nodes indicating GO terms (Hexagone) or succinylated proteins (Circle) and linked with the edge. Biological relevance and the involved proteins were interconnected. Relative expression of succinylation higher in RANKL is closer to the red colour.

A quantitative proteomic analysis of the succinylome determined 39 succinylated peptides in 19 proteins (Table S1). Interestingly, many of the detected proteins contain multiple succinylation sites (Fig. 4A). This includes six sites in serine hydroxymethyltransferase 2 (SHMT2), six sites in malate dehydrogenase 2 (MDH2), four sites in the ATP synthase subunit beta (ATP5B) and two sites in citrate synthase (CS) (Table S1). To determine the differentially succinylated proteins affected by RANKL treatment, the quantitative succinylome was processed with a two‐tailed Student's *t*‐test, *P* < 0.1 and |log_2_FC| >2 (Table S2). We plotted the significantly succinylated proteins from RANKL‐treated and unstimulated samples according to fold change (Fig. 4B). Interestingly, many succinylated lysine residues were found already in the untreated state, and their succinylation level decreased after treatment. The most notable decreases in RANKL‐related succinylation were observed in malate dehydrogenase (MDH2), the enzyme that converts malate to oxaloacetate in the TCA cycle, at K78 (−2.96 in log_2_FC) and the serine hydroxymethyltransferase (SHMT2), a key enzyme of the one carbon metabolism in the mitochondria, at K269 (−1.90 in log_2_FC) and K469 (−1.56 in log_2_FC) [34, 35].

To gain a better understanding of the physiological role of protein succinylation in osteoclast development, Gene Ontology (GO) analysis for subcellular localisation and biological processes was performed using ShinyGO [36]. Interestingly, all succinylated proteins were found to be localised in the mitochondria, predominately enriched in the mitochondrial nucleoid, myelin sheath and the mitochondrial matrix (Fig. 4C). These mitochondrial enzymes contribute to metabolic processes including the TCA cycle, dicarboxylic and metabolic processes, and aerobic respiration, respectively (Fig. 4D). Strikingly, the initiating enzyme of the TCA cycle, citrate synthase, was the only protein to show a statistically significant increase in succinylation after RANKL treatment, with an 8.48‐fold increase (3.08 in log_2_FC) at the K76 lysine residue (Fig. 4B). Additionally, CS was also found to be succinylated at K395 (Table S2); however, this succinylation site was only detected in two out of six biological replicates and was therefore not statistically relevant.

The combined results from GO analysis and succinylation intensity were comprehensively visualised in a network with Cytoscape app (Fig. 4E). Succinylated proteins are shown in circular nodes close to thick red colour when upregulated in RANKL and interconnected with biological processes in hexagon nodes. The network analysis shows distinct changes in succinylation intensity of targeted enzymes and clear interconnections within the energy metabolism of mitochondria. Our finding implies that RANKL modulates the regulation of mitochondrial metabolic pathways via protein succinylation to support osteoclastogenesis.

### Citrate synthase activity is modulated by RANKL

**Fig. 5:**
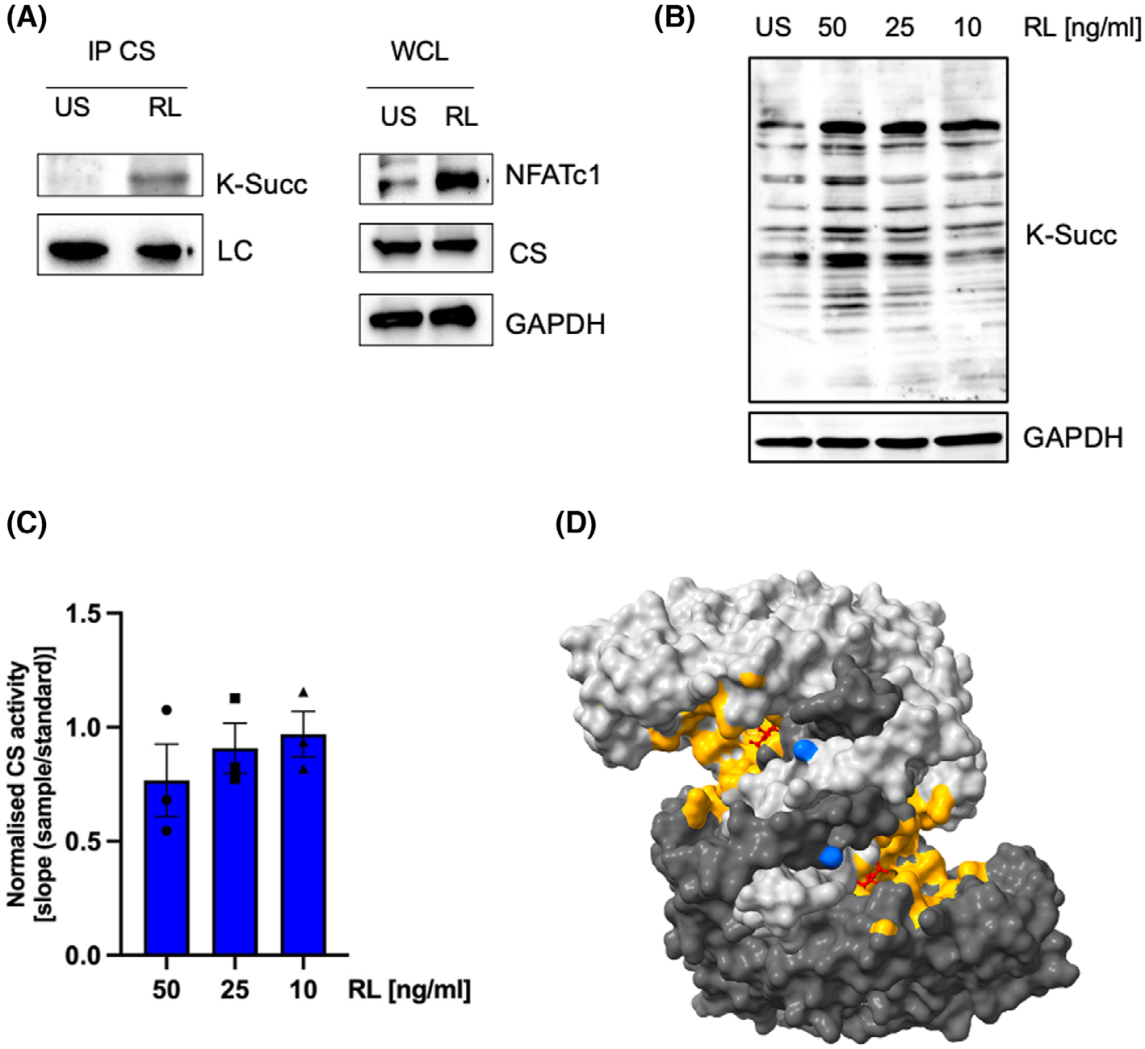
Succinylation of citrate synthase modulates its enzymatic activity. (A) Validation of the succinylation status of citrate synthase (CS) using a pan‐succinyl lysine antibody. RAW 264.7 cells were left unstimulated or stimulated with RANKL for 2 days. Endogenous citrate synthase from cell lysates was blotted with antisuccinyl lysine antibody. Light chain (LC) expression was used as a loading control for immunoprecipitation (IP). Whole cell lysates (WCL) indicated RANKL‐specific stimulation with NFATc1 and basal level of citrate synthase (*n* = 3). (B) Abundance of global protein succinylation according to RANKL concentration. RAW 264.7 cells were stimulated in a series of RANKL concentrations (10, 25, or 50 ng·mL^−1^) for 2 days. GAPDH was used as a loading control (*n* = 3). (C) Enzymatic activity of citrate synthase decreases with increasing RANKL concentration. Changes in absorbance per second (ΔA412/s) were plotted in the linear range to calculate the slope and calculated slopes were then normalised by performing a linear regression fit relative to the standard slope using porcine heart citrate synthase. Bar graphs represent means ± SEM. Differences between groups were statistically assessed using a one‐way ANOVA test (*n* = 3), however the changes remained statistically non‐significant. (D) Model of the citrate synthase structure in its open conformation. K76 is depicted in blue, the ligands (citrate and CoA) are shown as ‘ball‐and‐stick’ and the active site is marked in yellow. Structural visualisation was generated using UCSF ChimeraX version 1.4.

Next, we used a pan‐succinyl lysine antibody to verify the detected increase in succinylation of citrate synthase that had been found by MS by western blot analysis. RAW 264.7 cells were stimulated with RANKL for 2 days or left untreated before lysis and immunoprecipitation of citrate synthase. The left panel of Fig. 5A shows that RANKL treatment led to a strong increase in citrate synthase succinylation (Fig. S5A). As a control, the corresponding whole cell lysates (right panel) were tested for citrate synthase levels, which did not vary between untreated and RANKL stimulated cells. This shows that the observed increase in succinylation is indeed dependent on increased lysine succinylation and not caused by enhanced expression levels.

Next, we investigated whether increasing concentrations of RANKL would result in an increase in the detectable protein succinylation. Indeed, increasing RANKL concentrations ranging from 10 to 50 ng·mL^−1^ RANKL caused an observable increase in the overall succinylation pattern (Fig. 5B). To evaluate whether the succinylation state would modulate the enzymatic activity of citrate synthase, a citrate synthase assay was performed using increasing RANKL concentrations. For this, extracts were prepared from RAW 264.7 cells treated for 48 h with 10, 25 and 50 ng·mL^−1^ RANKL, respectively. Citrate synthase activity of osteoclasts differentiating under increasing RANKL concentrations was measured by recording a change in colour following the production of CoA‐S‐S‐TNB by recording the absorbance at 412 nm (Fig. 5C). The results for the slope of citrate synthase activity normalised to the standard (porcine citrate synthase) are presented as bar graphs and show that increasing concentrations of RANKL and thus increasing succinylation levels of citrate synthase decrease the activity of the enzyme. To understand how the succinylation status of citrate synthase could be regulated by succinylation, we visualised the localisation and accessibility of the lysine residue K76 in the available porcine protein structure of citrate synthase (Fig. 5D). Sequence homology to both the mouse and human versions of the protein was verified by performing a ClustalW alignment (Fig. S5B). The model suggests that K76 might change the ability of citrate synthase to move from the open to the closed state conformation when succinylated, which would negatively impact the ability to produce citrate (see also Videos [Link], [Link]). Citrate synthase is a critical gatekeeper for the TCA cycle because this metabolic circuit depends on the availability of citrate. Thus, it can be hypothesised that lower citrate levels caused by enhanced succinylation of CS should lead to a decrease in TCA cycle activity and a decrease in ATP production.

## Conclusion

ACOD1 expression is induced by RANKL and causes an accumulation of mitochondrial succinate, resulting in the succinylation of mitochondrial proteins during early osteoclast differentiation. Out of the identified set of proteins, citrate synthase was the only protein that showed succinylation only in the presence of RANKL. Subsequent experiments showed that CS succinylation lowers the activity of the enzyme, thus supporting an initial shift of differentiating osteoclasts towards glycolysis that is relieved through an increase of SIRT5 and a decrease in ACOD1 expression at later time points.

## Discussion

Osteoclast differentiation is characterised by a switch of metabolic pathways, where early osteoclastogenesis seems to require enhanced metabolism of glucose while oxidative phosphorylation seems to be indispensable for later stages of osteoclast differentiation [37]. Our data suggest that the RANKL‐mediated increase in ACOD1 expression and a late upregulation of SIRT5 are critical steps in this metabolic reprogramming. Citrate synthase seems to be a central node for RANKL‐mediated metabolic reprogramming as its enzymatic activity decreases as a consequence of enhanced succinylation. Whether this is caused by a decrease in enzymatic activity or through steric hindrance of the substrate entering the enzymatic cleft after succinylation still needs to be investigated. However, it can be seen that K76, which gets succinylated in response to RANKL treatment, is in close proximity to the enzymatic cleft. It can be hypothesised that as a consequence, TCA cycle activity decreases and ATP levels are lowered. Using diethyl succinate to mimic hypersuccinylation, we observed a decrease in osteoclast fusion but not numbers, pointing towards a late effect on osteoclastogenesis. This suggests that the switch towards oxidative phosphorylation through a reduction in protein succinylation is required for successful differentiation and fully functional osteoclasts. Blocking the mitochondrial complexes through rotenone (complex I), antimycin A (complex III) or oligomycin (complex V) was also described to decrease the speed of osteoclast formation [9]. However, the authors did not investigate the expression of specific genes or comment on the morphology of the obtained osteoclasts in their study. In an approach using the targeted inhibition of the ATP synthase β subunit, a decrease in bone erosion in a model of collagen‐induced arthritis was observed, again highlighting the fact that oxidative phosphorylation is required for the functionality of osteoclasts [38]. This is also supported by the finding that macrophages that lack AMPK1 show decreased mitochondrial activity and a switch towards fatty acid synthesis, which results in a phenotype of increased inflammation and excessive pro‐inflammatory cytokine production [18].

The catabolic destruction of glucose generates various CoA metabolites that can be attached to lysine residues. However, the functional consequences of these post‐translational modifications are not clear yet, and many data have been obtained from model systems investigating pathologic conditions such as cancer or through knockout models of SIRT5 that force the cell towards hyperacylation. It is possible that the resulting effects are therefore exaggerated and do not adequately display the physiologic impact of such modifications. In a recent paper, the impact of protein acylation on the function of cardiac mitochondria was therefore investigated. Interestingly, it was observed that such modifications had a comparatively low impact on the bioenergetic state of the cells and the observed decrease in ATP levels was app. 15% [39]. With respect to osteoclastogenesis, however, such a subtle change might still be sufficient to fine‐tune the activity of glycolysis versus oxidative phosphorylation during the different stages of osteoclast differentiation.

The regulation of citrate synthase activity by succinylation and the resulting impact on TCA cycle activity has been investigated in other models. It was consistently found that in the presence of increased SIRT5 levels, lower succinylation was observed that resulted in increased levels of ATP [40, 41]. Consequently, low SIRT5 expression levels increased the succinylation of citrate synthase and reduced its enzymatic activity [42, 43]. Hypersuccinylation of citrate synthase was, for example, found to suppress cancer cell proliferation and migration [41]. The production of citrate is not only needed for TCA cycle progression and oxidative phosphorylation, but it acts also as a precursor for lipid biosynthesis [44, 45]. The enzyme ATP‐citrate lyase (ACLY) converts citrate into Acetyl‐CoA, which can then get further modified to generate different classes of lipids, such as polyunsaturated fatty acids, cholesterol esters, eicosanoids or sphingolipids [46]. The sphingolipid phosphatidylethanolamine, for example, was found to be required specifically for osteoclast fusion [47], while changes in the levels of the phosphoglyceride phosphatidylserine resulted in an impairment of osteoclast formation and function [48] and the presence of additional saturated fatty acids enhanced osteoclast survival in a high‐fat diet mouse model [49]. Consequently, the inhibition of ACLY therefore suppressed osteoclast differentiation. Here, the authors argue that the missing acetyl‐CoA prevented histone acetylation, as RANKL treatment was found to trigger the translocation of ACLY into the nucleus [50]. Our own data and the model presented for the succinylation of K76 suggest that the succinylation of this residue could change the ability of citrate synthase to shift into its active, closed conformation due to steric hindrance by this bulky post‐translation modification, which would also affect the downstream activity of ACLY [25]. More detailed analysis will be needed to investigate this question. Succinylation of CS at K395, a residue that is also close to the enzymatic cleft, was detected only in the two replicates from the first batch. There are several possible explanations for why this modification was not consistently detected across all replicates. One possibility is biological variation, where small differences in cell growth conditions or cell age could influence succinylation levels. However, a more likely explanation relates to the technical limitations of the protocol. Given the low abundance of the succinylated peptides, variations in enrichment efficiency between batches performed at different times may have affected detection. Furthermore, even minor differences in LC–MS system performance could impact the detection of this peptide. While LC–MS is a powerful tool for identifying peptides and post‐translational modifications, it is important to remember that some peptides may fall below the detection limit, and thus, their presence in the sample may go undetected and unquantified.

In our study, we observed a strong and unexpected downregulation of mitochondrial serine hydroxymethyltransferase (SHMT2) succinylation. SHMT2 catalyses the rate‐limiting step of serine metabolism and is known to play an important role in the proliferation of cancer cells as it promotes metabolic reprogramming [34]. In other studies, hypersuccinylation of SHMT2 through SIRT5 inhibition had been described to decrease SHMT2 activity and tumour growth [51]. As there was no obvious link between SHMT2 and bone homeostasis or bone‐related diseases, we compared the expression of SHMT2 in RAW 264.7 cells and primary bone marrow‐derived macrophages (BMDM) and noticed that the cell line highly overexpresses SHMT2, suggesting that this result is an artefact caused by the choice of the model system (Fig. S6).

Moreover, most of the identified proteins displayed multiple succinyl lysine sites upregulated or downregulated by RANKL. This indicates that RANKL may act as a switch for controlling the succinylation status to regulate protein properties specific to osteoclast differentiation. It is also of note that many proteins were succinylated in the absence of RANKL treatment. This is most likely explained by the fact that RAW 264.7 macrophages continuously secrete M‐CSF into the supernatant, and the RANKL‐negative state can therefore not be considered as unstimulated. A question may arise regarding the much stronger succinylation levels for RANKL‐induced succinylation seen by Western blot analysis. To identify succinylated proteins specific to RANKL treatment by LC–MS/MS, dimethyl labelling reagents co‐targeting lysine (K) residues were tagged to samples prior to antibody enrichment and MS analysis. Dimethyl labelling might have affected the recognition of the succinylated peptides by the antibody used in PTMscan. Therefore, it is possible that more proteins are succinylated in the cell than reported in this study.

Mitochondria are central organelles that play a pivotal role in many cellular processes. Mitochondrial DNA can act as a danger associated molecular pattern (DAMP) that alerts the immune system to self‐damage. In the context of viral infections, mitochondria serve as platforms for innate immune signalling and mitochondrial dynamics regulate cellular immunological functions of T cells, macrophages and dendritic cells via oxidative phosphorylation and the production of ROS and various metabolites, such as succinate, itaconate or citrate [6]. While the pro‐inflammatory effects of succinate during infection are well‐described [5, 52], the effect of succinylation on the process of inflammation has not been investigated, although it was observed that LPS treatment leads to an increase in protein succinylation [14]. The data presented by Tannahill *et al*. show that LPS stimulation of primary macrophages targets proteins involved in protein chaperon folding, leukocyte mediated immunity, and regulation of several metabolic processes by succinylation. This pattern is distinctly different from what we observed for macrophages treated with RANKL, suggesting that the succinylation pattern is specifically targeting those enzymes that play a pivotal role in the respective activated signalling process.

## Materials and methods

### Reagents

Diethyl succinate (112402) and recombinant porcine citrate synthase (C3260‐1K4) from Sigma‐Aldrich (Taufkirchen, Germany), recombinant murine RANKL (416‐ML, Biotechne, Minneapolis, Minnesota, UK), ß‐mercaptoethanol (P07‐05020, PAN Biotech, Aidenbach, Bayern, Germany), LPS‐B5 Ultrapure (LPS from *E. coli* 055: B5, tlrl‐pb5lps, InvivoGen, CA, USA), Protease Inhibitor Mix G (39101), Phosphatase Inhibitor‐Mix II (39055), BlueBlock PF (10×) (42591) from SERVA (Heidelberg, Germany) and SIRT5 inhibitor MC3482 (TargetMol, Boston, Massachusetts, China).

### Cell lines and stimulation

RAW 264.7 cells (RRID:CVCL_0493) were purchased from ATCC/LGC Standards (Wesel, Germany). The cells were carefully maintained and passaged no more than 20 times to minimise the risk of mutation. Gene expression and surface markers were routinely evaluated over the past three years for authentication. The cells were also regularly tested for mycoplasma contamination using PCR‐based detection and were confirmed to be mycoplasma‐free culture conditions to ensure consistency and reliability of results. Cells were cultivated in high‐glucose Dulbecco's medium supplemented with 10% FCS and 1% penicillin/streptomycin. RAW 264.7 cells were differentiated into osteoclasts with 50 ng·mL^−1^ RANKL for 5 days. For stable cell proliferation, 50 μm ß‐mercaptoethanol was added to the culture medium. Other stimuli used for experiments include the following: 100 ng·mL^−1^ *E. coli* LPS and 5 mm diethyl succinate [53].

### Generation of BMDM

6‐ to 8‐week‐old female C57BL/6J (Janvier Labs, LeGenest St. Isle, France) mice were sacrificed in accordance with the animal care guideline approved by German animal welfare authorities under the project number T‐14/22. Housing and health status of the mice were monitored as recommended by the Federation of Laboratory Animal Science Associations (FELASA). Bone marrow cells were isolated and differentiated into BMDM as described previously [54]. BMDM were harvested on Day 6 and stimulated with 25 ng·mL^−1^ M‐CSF and 50 ng·mL^−1^ RANKL to obtain osteoclasts.

### TRAP staining

To analyse osteoclast differentiation, 5 × 10^3^ cells were seeded onto a 24‐well plate and stimulated with RANKL or the combination of RANKL and diethyl succinate for 5 days. On Day 3, half of the volume was refreshed. A commercial acid phosphatase and tartrate‐resistant acid phosphatase (TRAP) kit from Merck KGaA (Darmstadt, Germany) was used to fix and stain the cells on the day of harvesting. TRAP‐positive cells containing three or more nuclei were identified and counted as multi‐nucleated osteoclasts.

### Quantitative real‐time PCR

About 2.5–5 × 10^4^ RAW 264.7 macrophages or BMDM were seeded and stimulated in a 24‐well plate. Total RNA isolation was performed using the innuPREP Mini Kit from Analytik Jena (Jena, Germany). The Biozym cDNA Synthesis Kit was used to synthesise cDNA (Biozym Scientific GmbH, Hessisch Oldendorf, Germany). With the StepOne real‐time PCR machine (Applied Biosystems, Darmstadt, Germany), quantitative RT‐PCR was carried out using qPCRBIO Syber Green Mix Hi‐ROX (PCR Biosystems, London, UK). Following a 2 min at 95 °C initial denaturation step, there were 40 cycles at 95 °C for 5 s and amplification at 60 °C for 20 s. Primers were purchased from Biomers (Ulm, Germany) and a list of primers used can be found in Table 1.

**Table 1:** Summary of primer pairs used in experiments.

Gene	Sequence 5′ → 3′
*Rps29*	Fw: AGCCGACTCGTTCCTTTCTC Rv: CGTATTTGCGGATCAGACC
*Acp5*	Fw: TTCCAGGAGACCTTTGAGGAc Rv: GGTAGTAAGGGCTGGGGAAG
*Atp6v0d2*	Fw: TCAGATCTCTTCAAGGCTGTGCTG Rv: GTGCCAAATGAGTTCAGAGTGATG
*Ctsk*	Fw: AGGGAAGCAAGCACTGGATA Rv: GCTGGCTGGAATCACATCTT
*Dcstamp*	Fw: AAAACCCTTGGGCTGTTCTT Rv: GTTCCTTGCTTCTCTCCACG
*Nfatc1*	Fw: CAGGGCTCACTATGAGACGG Rv: AGCTGTAGCGTGAGAGGT
*Ocstamp*	Fw: TGGGCCTCCATATGACCTCGAGTAG Rv: TCAAAGGCTTGTAAATTGGAGGAGT
*Mmp9*	Fw: CAGCCGACTTTTGTGGTCTTC Rv: CGGTACAAGTATGCCTCTGCCA
*Sirt5*	Fw: GTTCGCTGTCTCCAGGTTATT Rv: AAAGCCACCTGGCAAAACTG
*Shmt2*	Fw: CCCCTATGTTCCGCGAGTAC Rv: GTTGGCTGTGATGGAGACGA

### Mitochondrial fractionation

For biochemical fractionation of mitochondria, 5 × 10^6^ RAW 264.7 cells were plated on a 10 cm culture dish and stimulated with M‐CSF, M‐CSF/RANKL, or *E. coli* LPS for 24 h. Mitochondrial Isolation was performed using the Mitochondria Isolation Kit for Mammalian cells (Thermo Fisher Scientific, MA, USA). According to the manufacturer's protocol, cells were collected by scraping in Solution A from the kit supplemented with a protease inhibitor Mix G (20×). Cell suspensions were homogenised with 30 strokes using a glass Dounce Homogeniser (Sigma‐Aldrich). Mitochondrial and cytosolic fractions were obtained by sequential centrifugation steps at 4 °C. Initially, homogenates were centrifuged for 10 min at 900 ***g***, followed by an additional centrifugation for 15 min at 13800 ***g***. Cytosolic samples were obtained in this step. The pellet was resuspended in CHAPS buffer supplemented with a protease inhibitor cocktail. Supernatants containing the mitochondrial fraction were isolated after centrifugation for 2 min at 16200 ***g***.

### Western blot analysis

For western blot analysis, 1 × 10^6^ RAW 264.7 cells were seeded in a six‐well plate. Lysis was performed using RIPA buffer supplemented with protease inhibitors (SERVA) as described previously [55]. Protein lysates were further quantified by a bicinchoninic acid (BCA) assay from Cyanagen Srl (Bologna, Italy). About 10 μg of lysates were separated on 4–20% gradient SDS/PAGE gels (Anamed, Gross‐Bieberau, Germany) for 1 h before semi‐dry Western blot transfer to nitrocellulose membranes. Subsequently, the membrane was blocked with 1 × Blue Block blocking buffer (SERVA, Heidelberg, Germany). The primary antibody was diluted in Blue Block buffer according to the manufacturer's recommendation, and membranes were incubated overnight at 4 °C. Protein bands of interest were identified by chemiluminescence (Intas Science Imaging, Göttingen, Germany) after an hour of incubation with the corresponding secondary antibody. LabImage software (KAPELAN Bio‐Imaging GMBH, Leipzig, Germany) was used to quantify western blot data. The following antibodies used are described in Table 2.

**Table 2:** List of antibodies used in western Blot experiments.

Antibody	Company	Identifier
Rabbit polyclonal anti‐Acod1	Cell Signaling	17 805
Mouse monoclonal anti‐NFATc1	BD Biosciences	556 602
Mouse monoclonal anti‐GAPDH	Proteintech	60 004‐1‐Ig
Mouse monoclonal anti‐Succinyllysine	PTM Biolabs Inc.	PTM‐419
Rabbit polyclonal monoclonal anti‐Hsp90	Cell Signaling	4874
Rabbit polyclonal anti‐CoxIV C	Cell Signaling	4850
Rabbit monoclonal anti‐Sirt5	Cell Signaling	8779
Mouse monoclonal anti‐citrate synthase	Santa Cruz	sc‐390 693
Rabbit monoclonal anti‐β‐Actin	Proteintech	20 536‐1‐AP
Goat monoclonal anti‐mouse IgG, HRP‐linked	Cell Signaling	7076
Goat monoclonal anti‐Rabbit IgG, HRP‐linked	Cell Signaling	7074
Goat polyclonal anti‐mouse IgG light chains	Jackson/Dianova	115–035‐174

### Immunoprecipitation

In total, 1.5 × 10^7^ RAW 264.7 cells were seeded onto a 10‐cm dish and stimulated with RANKL for 2 days. Cells were lysed in Brij97 buffer supplemented with a protease inhibitor cocktail. Obtained total lysates were incubated with protein A/G plus agarose beads and an antibody against citrate synthase by overnight rotation at 4 °C. Beads were gently washed twice with Brij buffer and once with TNE buffer for 15 s of each step. Samples were prepared in 2× SDS loading buffer and boiled for 2 min at 95 °C. Supernatants collected after centrifugation at 15 000 rpm for 2 min were subjected to western blot analysis. Specific light chain‐HRP antibody was used as a loading control for the input of the pull‐down antibody.

### Succinate measurement

To quantify intracellular succinate levels, 0.5 × 10^6^ RAW 264.7 cells were left unstimulated or treated with RANKL and *E. coli* LPS, respectively, for 2 days in a six‐well plate. Cells were lysed in water and sonicated with three ultrasound bursts of 30 s each. Supernatants were collected after centrifugation at 4 °C for 10 min at 15,000 rpm. BCA assay was performed to obtain about 30–50 ng of protein lysates. According to the manufacturer's instructions, intracellular succinate was measured using the EnzyChromTM succinate assay kit (BioAssay Systems). Succinate standards were made in a range from 0 to 40 μm. The working solution was equally distributed to samples and standards and incubated for 30 min at RT. The reaction was measured by reading a fluorescence excitation and emission wavelength between 530 nm and 585 nm using a CLARIOstar (BMG LABTECH GmbH, Ortenberg, Germany). The slope from the succinate standard was determined and the succinate concentration of the samples was calculated.

### Sample preparation for PTMscan analysis

3 × 10^6^ RAW 264.7 cells were seeded onto a 10‐cm dish and were either stimulated with 50 ng·mL^−1^ RANKL or left unstimulated for 2 days. Protein lysates were prepared using 8 m urea‐containing buffer. A total of 1–2 mg protein was precipitated using the adapted Wessel‐Flügge method [56]. After reduction and alkylation for 30 min at RT using 10 mm TCEP and 40 mm CAA, samples were digested using trypsin at a 1:100 enzyme:protein ratio and incubated overnight at 37 °C.

Desalting and dimethyl labelling of the peptides were performed on SepPak C18 columns (Waters WAT036820). Briefly, each SepPak C18 column was conditioned with ACN followed by a single wash with 50% ACN/0.1% TFA. Acidified peptides were loaded on each SepPak C18 column. Immobilised peptides were flushed with light labelling reagent (100 mm TEAB pH 8.0, 4% formaldehyde, and 0.6 m cyanoborohydride) or medium labelling reagent (100 mm TEAB pH 8.0, 4% deuterated formaldehyde, and 0.6 m cyanoborohydride) for at least 10 min. Peptides for each condition were eluted using 80% ACN/0.1% TFA. Two related samples, light and medium labelled, were combined and completely dried overnight. PTMscan kit #13764 (Cell Signalling Technology) was used for the enrichment of succinylated peptides. The manufacturer's protocol was followed with minor changes. Shortly, peptides were mechanically resuspended in 1× IAP buffer. The neutral pH was confirmed using pH indicator paper and a pH adjustment was not needed. Peptide solution was centrifuged for 5 min at 10 000 ***g*** and 4 °C. Beads were washed three times with PBS and finally resuspended in 30 μL of PBS. Peptide solution was transferred into the vial containing the beads and incubated on a rotator wheel for 2 h at 4 °C. After this time, beads were washed twice with IAP buffer and three times with HPLC‐grade water. Finally, peptides were eluted from the beads twice using 0.15% TFA. Digested proteins were desalted on self‐made C18 Empore® extraction discs (3 m, Maplewood, MN) STAGE tips [57].

### LC–MS/MS analysis

Samples were suspended in 0.1% TFA and analysed using an Ultimate 3000 liquid chromatography system coupled to an Orbitrap QE HF (Thermo Fisher Scientific) as described before [58]. The MS/MS spectra were searched against the Swiss‐Prot *Mus musculus* (UP000000589, 21959 sequences) protein database and a customised contaminant database (part of MaxQuant, MPI Martinsried) using Proteome Discoverer 2.5 with Sequest HT (Thermo Fisher Scientific). A fragment ion mass tolerance was set to 0.02 Da and a parent ion mass tolerance to 10 ppm. Trypsin was specified as the enzyme. Carbamidomethylation was set as a fixed modification of cysteine. Dimethylation (2plex) was set as the quantification method. The following modifications were set as dynamic: oxidation (methionine), deamidation (asparagine, glutamine), dimethylation (lysine), succinylation (lysine), acetylation (N terminus), methionine loss (N terminus) and methionine loss and acetylation (N terminus). Peptide quantification was done using the precursor ion quantifier node with the Top N Average (*n* = 3) method set for protein abundance calculation [56].

### PTMscan data processing and GO analysis

Statistical analysis was conducted and visualised with a volcano plot using an R package. Protein abundances were logarithmically transformed to log_2_ values, followed by analysis using a two‐tailed Student's *t*‐test, where log_2_(fold‐change) >1 at −log(*P*‐value) >1 indicated significant succinylated proteins induced by RANKL compared to the unstimulated control.

GO‐term enrichment analysis was performed by ShinyGO for cellular component and biological process with FDR (false discovery rate) set to 0.05 [36]. Using the STRING web database v12.0 (https://string‐db.org/), we classified succinylated proteins in both experimental conditions according to the GO categories within the biological process domain. An interaction confidence score of ≥0.40 was applied to identify biological networks among related genes, which were subsequently visualised using cytoscape v3.10.0. In the generated network, nodes represented GO terms (hexagons) and succinylated proteins (circles), with edges illustrating the interactions between them. The biological relevance of these interactions and the involved genes was examined. The degree of red and blue coloration in the nodes indicated the abundance of succinylated proteins, with red signifying upregulation and blue indicating downregulation in response to RANKL stimulation.

### Citrate synthase activity assay

Sources of reagents used for this experiment were all obtained from Sigma‐Aldrich [59]. 3 × 10^6^ RAW 264.7 cells were seeded onto a 10‐cm cell culture dish and stimulated with 10, 25, and 50 ng·mL^−1^ RANKL for 2 days. Cells were lysed in water and rapidly frozen in liquid nitrogen to store at −80 °C until replicates were prepared. To obtain protein lysates, cells underwent three freeze–thaw cycles using liquid nitrogen. During the entire sample preparation, samples were kept on ice. Supernatants were obtained after centrifugation for 10 min at 15 000 rpm. BCA was performed to obtain 30 μg of protein lysates. Citrate synthase was diluted with a 1:5000 in 0.1 m Tris‐HCl buffer pH 7.0 and 5 μL was used as a positive control. H_2_O was used as a negative control. Reagents or buffers used for this assay were freshly made every month and kept at 4 °C. The enzymatic reaction CoA‐SH + DTNB → TNB + CoA‐S‐S‐TNB (*λ* = 412 nm) produces a yellow‐coloured product that can be quantified by the spectrophotometer. The detailed protocol is described by Eigentler *et al*. [59]. For optimal reaction, the machine was pre‐warmed to 30 °C and the absorbance at 412 nm was measured for 15 to 30 min with 30 s of interval time. Changes in absorbance per second (ΔA412/s) were plotted in the linear range to calculate the slope within a kinetic range (1–13 min). The calculated slopes were then normalised by performing a linear regression fit relative to the standard slope obtained using porcine heart citrate synthase (425 units·mg^−1^).

### Modelling of the citrate synthase structure

For pictures and videos, the program UCSF chimerax version 1.4 [60, 61] and the pdb files published by Remington *et al*. were used [62].

### Statistical analysis

Data from fluorescent and colorimetric analyses were analysed using the CLARIOstar software. Plotting graphs and further statistical analyses were performed using GraphPad Prism 10.0. Statistical values are described in each figure with *(*P* ≤ 0.05), **(*P* ≤ 0.01), ***(*P* ≤ 0.001), ****(*P* ≤ 0.0001); ns means not significant. Mass spec raw files were generated with the collaborative work with the Core Facility for Mass Spectrometry & Proteomics, Heidelberg University, and the proteomic data was analysed as indicated below.

## Conflict of interest

All authors declare that there is no conflict of interest.

## Author contributions

Methodology, validation and investigation (DY, YG and ES); validation, analysis, and resources (ML, TR, TH and MB); conceptualisation, writing and supervision (KFK).

## Supporting information


**Fig. S1.** RANKL stimulation enhances protein succinylation in RAW 264.7 cells and primary bone marrow‐derived macrophages.
**Fig. S2.** Differential expression of ACOD1 and SIRT5 modulate osteoclastogenic gene expression.
**Fig. S3.** Diethyl succinate modulates osteoclastogenesis in RAW 264.7 cells and primary bone marrow‐derived macrophages.
**Fig. S4.** PTMscan analysis approach.
**Fig. S5.** Citrate synthase is a target of succinylation.
**Fig. S6.** Basal mRNA levels of *Shmt2* in BMDMs and RAW264.7 cells.
**Table S1.** Identified succinylated peptides.
**Table S2.** Results of the statistical analysis to determine RANKL‐induced changes.

**Video S1.** Structural model of citrate synthase moving between open and closed conformation.

**Video S2.** Structural model of citrate synthase in the closed conformation.

**Video S3.** Structural model of citrate synthase in the open conformation.

## Data Availability

The mass spectrometry proteomics data have been deposited to the ProteomeXchange Consortium via the PRIDE partner repository with the dataset identifier PXD060675.
