# Supplementary material for: Protein succinylome analysis identifies citrate synthase as a central regulator of osteoclast metabolic activity
Source: FEBS J. 2025 Apr 2;292(14):3736–54. doi: 10.1111/febs.70090 (PMC12265861; doi:10.1111/febs.70090)
Supplement: Supplementary file 1 — Fig. S1. RANKL stimulation enhances protein succinylation in RAW 264.7 cells and primary bone marrow‐derived macrophages. Fig. S2. Differential expression of ACOD1 and SIRT5 modulate osteoclastogenic gene expression. Fig. S3. Diethyl succinate modulates osteoclastogenesis in RAW 264.7 cells and primary bone marrow‐derived macrophages. Fig. S4. PTMscan analysis approach. Fig. S5. Citrate synthase is a target of succinylation. Fig. S6. Basal mRNA levels of Shmt2 in BMDMs and RAW264.7 cells. Table S1. Identified succinylated peptides. Table S2. Results of the statistical analysis to determine RANKL‐induced changes. [file FEBS-292-3736-s004.pdf]

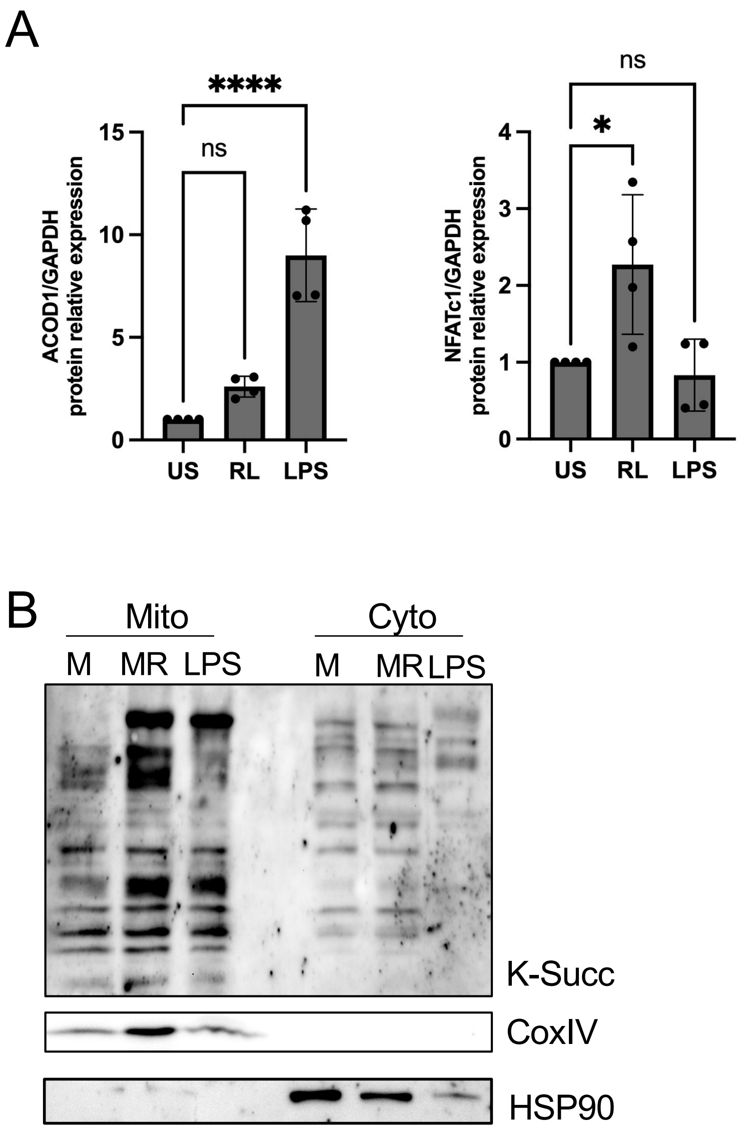

Figure S1: (A) Quantification of Western blots shown in Figure 1A (n=4). The lanes were quantified relative to the loading control and normalized to the untreated sample using the LabImage software. Statistical differences between groups compared to the untreated control were calculated by ordinary one-way ANOVA (* p≤0.05 and **** p≤0.0001). **(B)** Western blot analysis of the global protein succinylation in cell lysates obtained from the mitochondrial and cytosolic fraction of bone-marrow-derived macrophages. BMDMs were stimulated with M-CSF, M-CSF/RANKL (RL), and *E. coli* LPS for 24 h. HSP90 and CoxIV were used as cytosolic and mitochondrial markers, respectively. Statistical differences between groups compared to the untreated control were calculated by ordinary one-way ANOVA (* p≤0.05 and **** p≤0.0001).

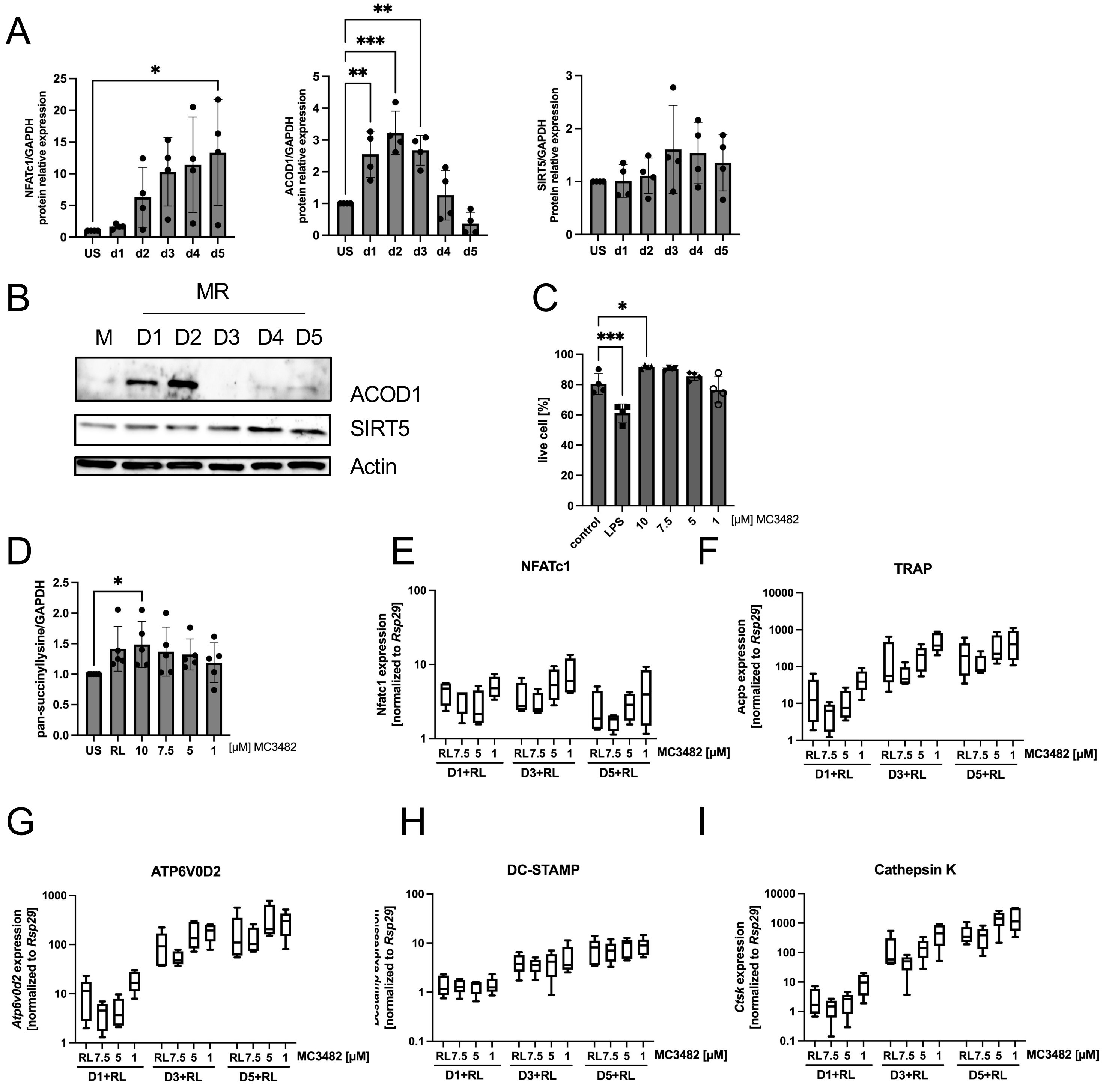

Figure S2: (A) Quantification of Western blots shown in Figure 2A (n=4). The lanes were quantified relative to the loading control and and normalized to the untreated sample using the LabImage software. **(B)** Western blot analysis of ACOD1 and SIRT5 expression in BMDM after RANKL stimulation, Actin was used as a loading control. **(C)** SYTOX™ viability assay. RAW 264.7 cells treated for 24 h with MC3482 (10, 7.5, 5 and 1 µM) were incubated with 300 nM SYTOX™ Green for 10 min (n=5). The percentage of the FITC negative population is shown as live cells. 1 µg/mL LPS was used as a control for reduced cell viability. **(D)** Quantification of the Western blot shown in Figure 2B (n=4). The lanes were quantified relative to the loading control and normalized to the untreated sample using the LabImage software. **(E-I)** Transcriptional levels of osteoclast fusion markers on days 1, 3 and 5 from cells stimulated with RANKL and different concentrations (7.5 µM, 5 µM, 1 µM) of the SIRT5 inhibitor MC3482. The relative gene expression was normalized with *Rsp29.* The fold gene induction was normalized with the DMSO treated control sample. For all experiments, Statistical differences compared to the untreated controls were calculated for all experiments by ordinary one-way ANOVA . *$p \le 0.05$, **$p \le 0.001$ and ***$p \le 0.0005$.

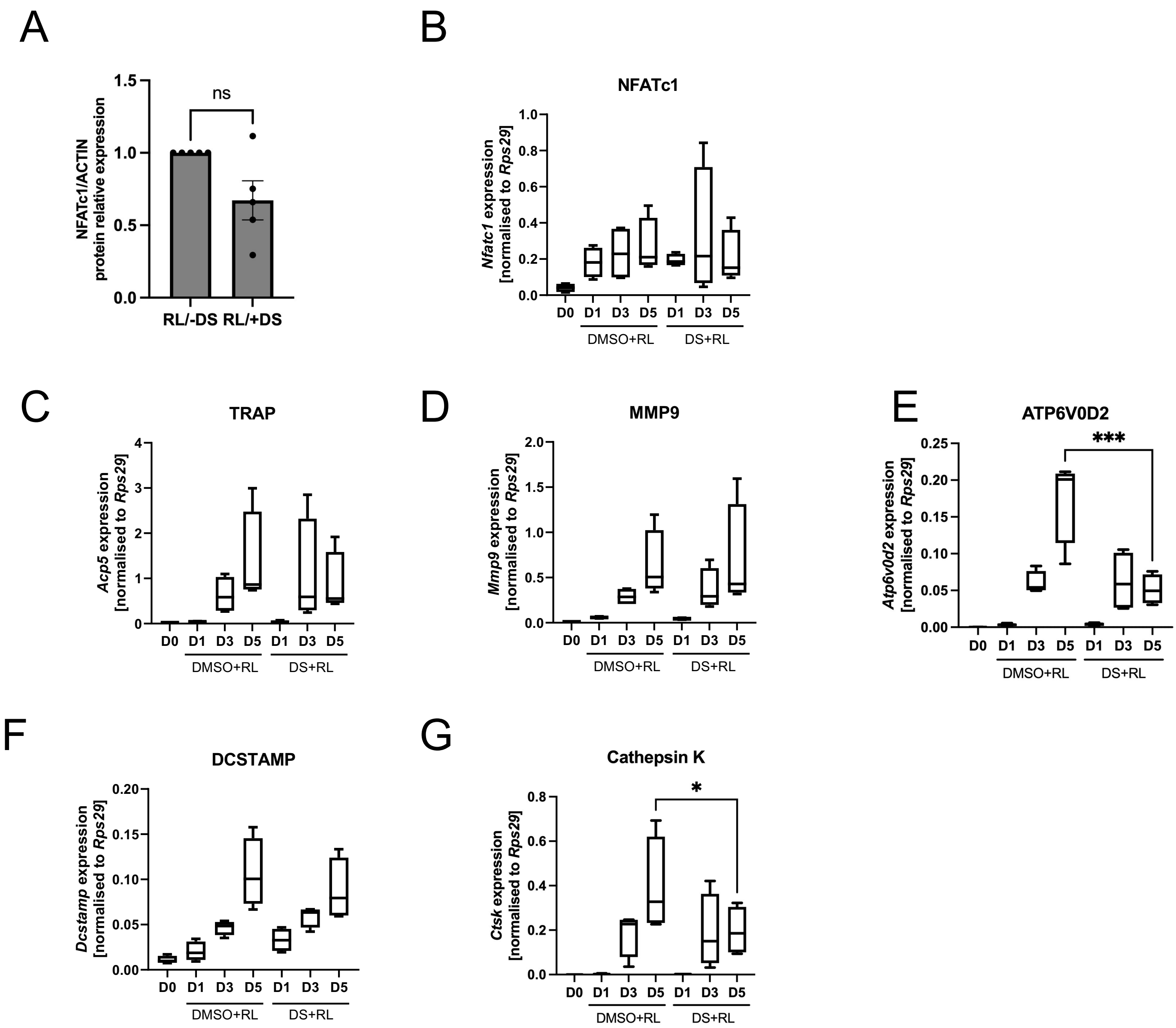

Figure S3: (A) Quantification of Western blots shown in Figure 3E (n=4). The lanes were quantified relative to the loading control and normalized to the untreated sample using the LabImage software. Statistical differences were calculated using a two-tailed t-test. **(B-G)** RT-PCR original data for gene expression on days 1, 3 and 5 from RAW 264.7 cells stimulated with RANKL or RANKL and diethyl succinate (5 mM) (n=4). The relative gene expressions were normalised to *Rps29.* Statistical differences between groups were calculated by ordinary one-way ANOVA between treatment groups at the same day (n=4). * p<0.05 , *** p<0.001.

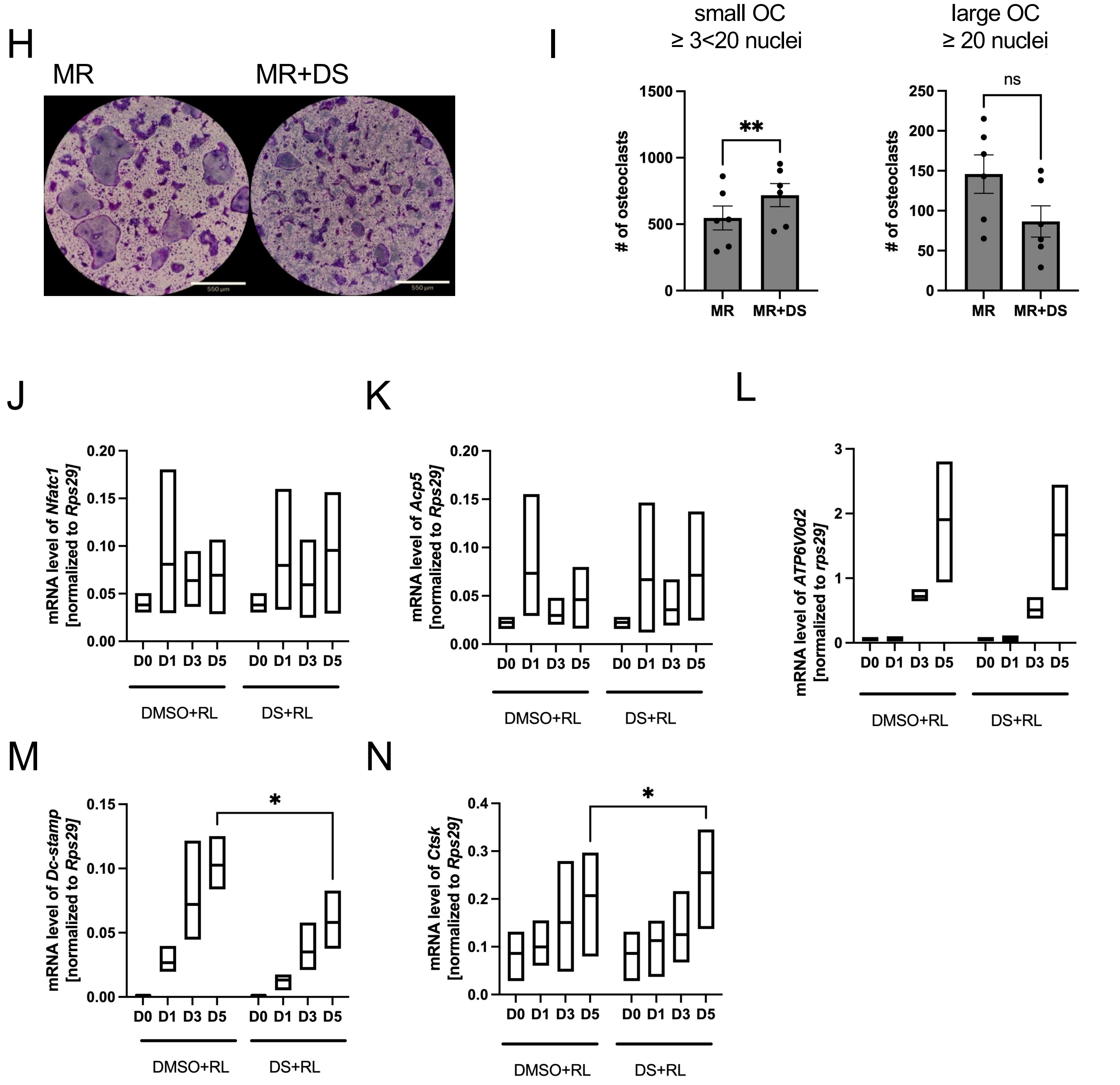

Figure S3: H) TRAP staining of multi-nucleated BMDM-derived osteoclasts in the presence of cell-permeable diethyl succinate (DS). During M-CSF/RANKL-derived osteoclast differentiation, BMDMs were cultured with 5 mM DS or DMSO as a solvent control.TRAP-positive cells were stained on day 6. Scale bar represents 550 µm. **I)** Quantification of TRAP-positive multi-nucleated cells upon DMSO+MR or DS+MR exposure (mean and SEM). Left panel depicts osteoclasts with ≥3 nuclei considered as regular osteoclasts (left) and osteoclasts with ≥20 nuclei as large osteoclasts (right). Statistical differences were calculated using a two-tailed t-test with **p<0.01. **(J-N)** RT-PCR original data for gene expression in BMDM on days 1, 3 and 5 from cells stimulated with RANKL or RANKL and diethyl succinate (5 mM) (n=3). The relative gene expressions were normalised to *Rps29.* Statistical differences between groups were calculated by ordinary one-way ANOVA between treatment groups at the same day. * p<0.05.

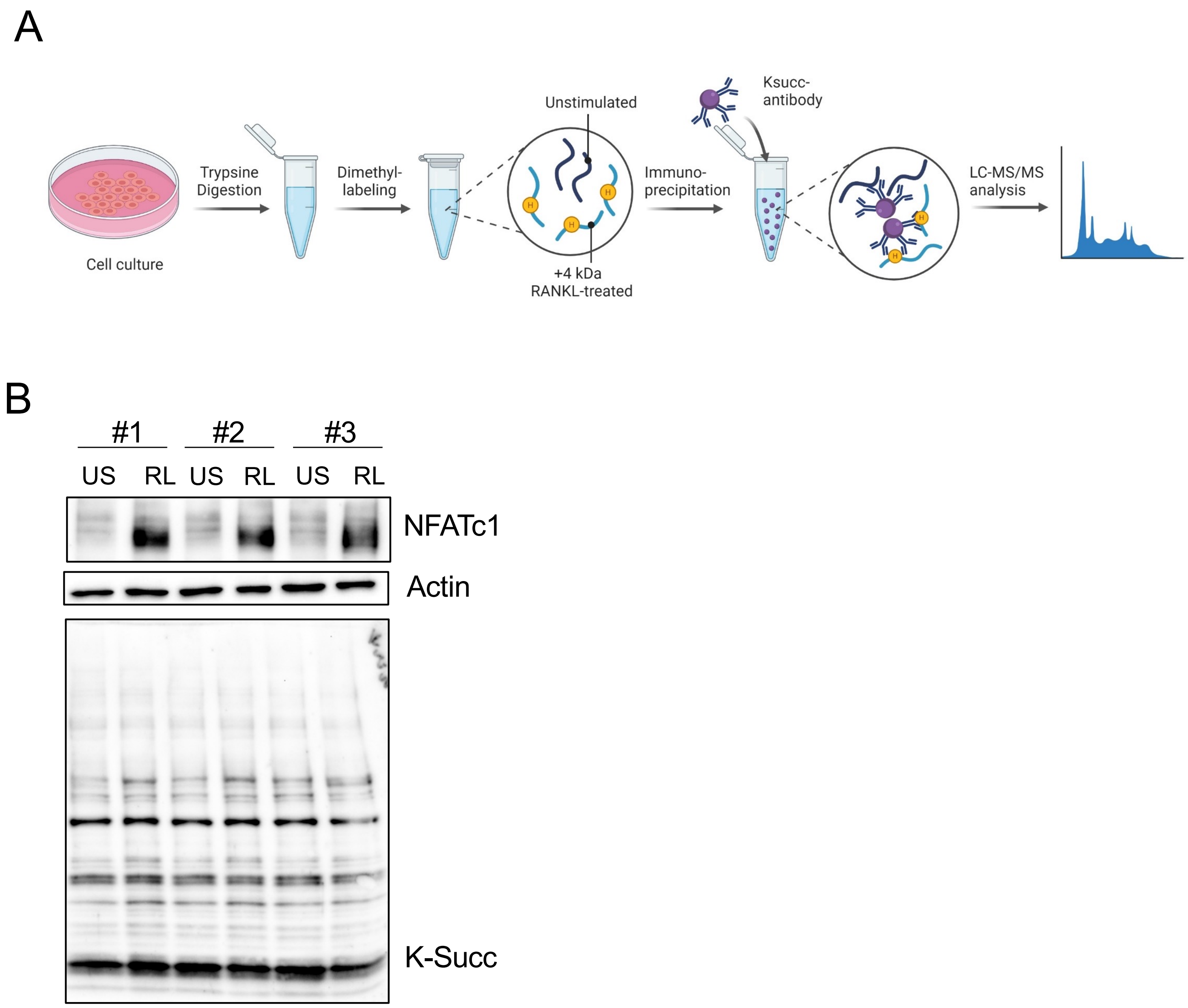

Figure S4. (A) Scheme of sample preparation for PTMscan analysis. RAW 264.7 cells were left unstimulated (US) or treated with RANKL for 48 h before lysis in a urea-containing buffer. A total of 1 mg protein was digested, and the resulting peptides were labelled using dimethyl labelling prior to a PTMscan enrichment and LC-MS/MS analysis. **(B)** Intensity of the whole cell protein succinylation. RAW 264.7 cells treated with RANKL for 48 hours and untreated cells were collected from the three independent experiments. As a control, global protein succinylation and abundance of NFATc1, a marker for osteoclast differentiation, were assessed by Western blot analysis using specific antibodies. Actin was used as loading control.

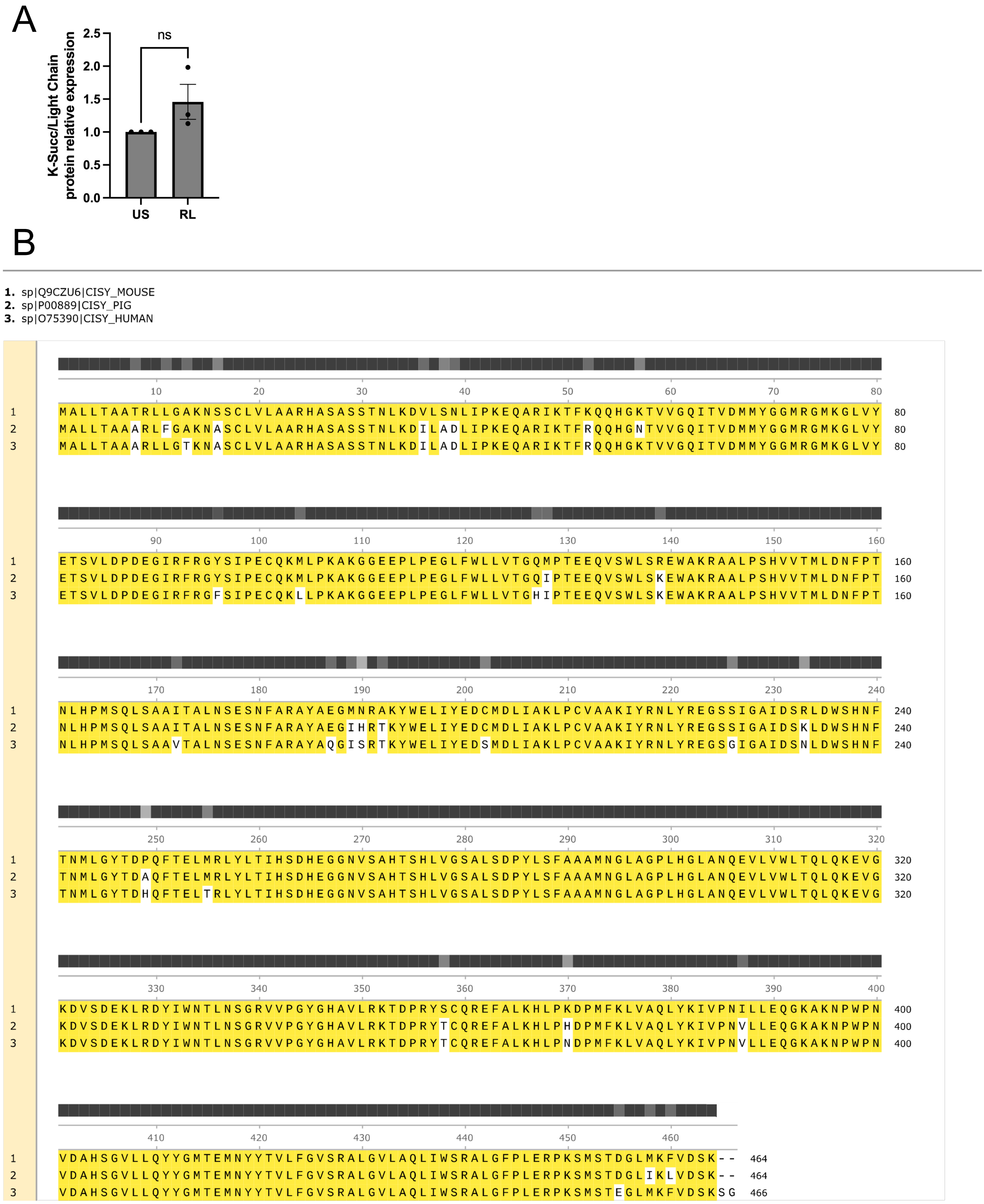

Figure S5. Clustal W ALignment

(A) Quantification of Western blots shown in Figure 5A (n=4). The lanes were quantified relative to the loading control and normalized to the untreated sample using the LabImage software. Statistical analysis was done using a two-tailed Student's t-test analysis, but differences remained statistically unsignificant. **(B)** The sequences of murine, human and porcine (*Sus scrofa*) citrate synthase were aligned for homology using Clustal W.

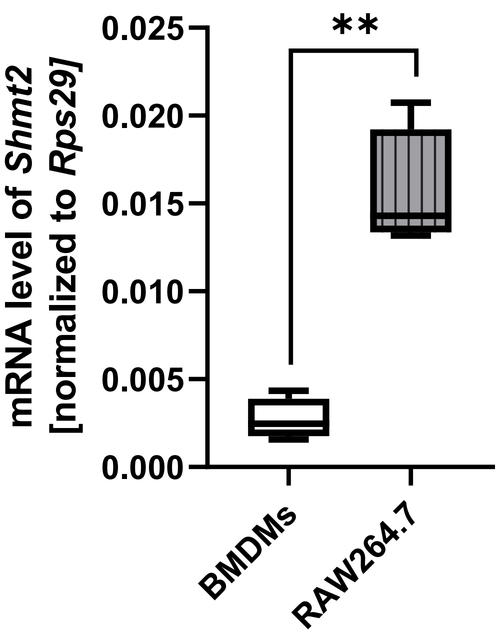

Figure S6. Basal mRNA levels of *Shmt2* in BMDMs and RAW264.7 cells. Each cell was directly collected from culture. Floating boxes represent line at median. Statistical differences between groups were calculated by a two-tailed Student's t-test, n=4, **p<0.01.

Table S1. Identified succinylated peptides.

Sequence	Modifications	Master Protein Accessions	Control_1	RANKL_1	Control_2	RANKL_2	Control_3	RANKL_3	Control_4	RANKL_4	Control_5	RANKL_5	Control_6	RANKL_6
AFFKGAWSNVLR	1xDimethyl:2H(4) [N-Term]; 1xSuccinyl [K4(100)]	P51881	NA	NA	NA	NA	17,38318	19,13322	NA	NA	NA	NA	NA	NA
ALKIPAMTIAK	1xDimethyl [K11]; 1xDimethyl [N-Term]; 1xSuccinyl [K3(100)]	P63038	NA	NA	NA	NA	19,04335	19,33948	NA	NA	NA	NA	NA	NA
ANTFVAELKGLDPAR	1xDimethyl:2H(4) [N-Term]; 1xSuccinyl [K9(100)]	P08249	NA	NA	NA	NA	16,43894	18,65881	NA	NA	NA	NA	NA	NA
ANVKGYLGPEQLPDCLK	1xCarbamidomethyl [C15]; 1xDimethyl [K17]; 1xDimethyl [N-Term]; 1xSuccinyl [K4(100)]	P08249	NA	NA	NA	NA	NA	NA	17,2573	13,76659	18,81644	15,16713	15,75998	15,11015
AQFEGIVTDLIKR	1xDimethyl [N-Term]; 1xSuccinyl [K12(100)]	P38647	16,87558	16,89666	17,95359	18,55035	19,34497	20,40042	17,40612	14,50683	14,71989	14,01378	17,44391	14,49071
CEFQDAYVLLSEKK	1xCarbamidomethyl [C1]; 1xDimethyl [K14]; 1xDimethyl [N-Term]; 1xSuccinyl [K13(99.7)]	P63038	NA	NA	NA	NA	16,76829	16,97827	NA	NA	NA	NA	NA	NA
DFLAGGVAAAISKTAVAPIER	1xDimethyl [N-Term]; 1xSuccinyl [K13(100)]	P51881	NA	NA	NA	NA	15,64505	20,44702	19,75541	18,58995	18,87179	18,14027	19,73062	19,35954
DGVTVAKSIDLK	1xDimethyl [K12]; 1xDimethyl [N-Term]; 1xSuccinyl [K7(100)]	P63038	NA	NA	NA	NA	16,93844	17,12827	NA	NA	NA	NA	NA	NA
DVFLPKPSWGNHTPIFR	1xDimethyl:2H(4) [N-Term]; 1xSuccinyl [K6(100)]	P05202	NA	NA	NA	NA	17,38917	18,97744	NA	NA	NA	NA	NA	NA
DVKLAQFIEK	1xDimethyl [K10]; 1xDimethyl [N-Term]; 1xSuccinyl [K3(100)]	Q9CZL5	NA	NA	NA	NA	18,56372	18,11253	NA	NA	NA	NA	NA	NA
EFSVYMTKDGR	1xDimethyl [N-Term]; 1xSuccinyl [K8(100)]	P05202	NA	NA	NA	NA	19,00783	19,43568	NA	NA	NA	NA	NA	NA
EGNDLYHEMIESGVINLKDATSK	1xDimethyl [K23]; 1xDimethyl [N-Term]; 1xSuccinyl [K18(100)]	P56480	NA	NA	NA	NA	15,61224	16,9999	NA	NA	NA	NA	NA	NA
ETGVDLTKDNMALQR	1xDimethyl [N-Term]; 1xSuccinyl [K8(100)]	P38647	NA	NA	NA	NA	17,93303	16,87446	NA	NA	NA	NA	NA	NA
FITALKSTGLR	1xDimethyl [N-Term]; 1xSuccinyl [K6(100)]	D3Z7P3	16,93791	16,47579	18,06801	15,01454	NA	NA	NA	NA	NA	NA	NA	NA
GASIVEDKLVEDLK	1xDimethyl [K14]; 1xDimethyl [N-Term]; 1xSuccinyl [K8(100)]	P26443	NA	NA	NA	NA	17,41352	15,64787	NA	NA	NA	NA	NA	NA
GKISVNDFIIK	1xDimethyl:2H(4) [N-Term]; 1xDimethyl:2H(4) [K11]; 1xSuccinyl [K2(100)]	Q8BMF4	NA	NA	NA	NA	16,12842	17,35997	NA	NA	NA	NA	NA	NA
GMKGLVYETSVLDPDEGIR	1xDimethyl:2H(4) [N-Term]; 1xSuccinyl [K3(100)]	Q9CZU6; Q80X68	16,15054	17,10366	15,38256	17,57019	15,5349	20,87515	16,11394	17,27022	14,43834	17,88855	16,31943	17,66756
GMKGLVYETSVLDPDEGIR	1xOxidation [M2]; 1xDimethyl:2H(4) [N-Term]; 1xSuccinyl [K3(100)]	Q9CZU6; Q80X68	16,44119	17,06161	15,11181	17,26002	15,84152	18,37742	15,61939	15,91522	14,75482	16,27171	15,65919	14,22471
GQKVLDSGAPIK	1xDimethyl [K12]; 1xDimethyl [N-Term]; 1xSuccinyl [K3(100)]	P56480	NA	NA	NA	NA	18,26678	18,17762	NA	NA	NA	NA	NA	NA
GQKVLDSGAPIKIPVGPETLGR	1xDimethyl [K12]; 1xDimethyl [N-Term]; 1xSuccinyl [K3(100)]	P56480	16,81741	17,1779	17,92034	17,45141	NA	NA	NA	NA	NA	NA	NA	NA
IKIIAPPER	1xDimethyl [N-Term]; 1xSuccinyl [K2(100)]	Q8BFZ3; P60710; P68134	NA	NA	NA	NA	19,17672	15,84541	NA	NA	NA	NA	NA	NA
ILHESTQTDKALYNR	1xDimethyl [N-Term]; 1xSuccinyl [K10(100)]	Q3V3R1	NA	NA	NA	NA	17,34869	15,97206	NA	NA	NA	NA	NA	NA
IMGLDLPDGGHLTHGYMSDVKR	1xOxidation [M2]; 1xDimethyl:2H(4) [N-Term]; 1xSuccinyl [K21(100)]	Q9CZN7	NA	NA	NA	NA	15,71751	15,5799	NA	NA	NA	NA	NA	NA
ITPFEEKMIAEAIPELK	1xDimethyl [K17]; 1xDimethyl [N-Term]; 1xSuccinyl [K7(100)]	P08249	NA	NA	NA	NA	16,49692	16,75847	NA	NA	NA	NA	NA	NA
IVPNILLEQGKAK	1xDimethyl [K]; 1xDimethyl [N-Term]; 1xSuccinyl [K]	Q9CZU6	16,94037	17,09634	18,34704	18,29486	NA	NA	NA	NA	NA	NA	NA	NA
LQDFKSFLLKDPETSQR	1xDimethyl [K10]; 1xDimethyl [N-Term]; 1xSuccinyl [K5(100)]	Q9CZN7	18,74808	18,51845	19,65044	19,14292	20,31967	17,12203	18,3971	14,74575	17,6744	14,46401	18,36898	14,4103
LVPGWTKPITIGR	1xDimethyl [N-Term]; 1xSuccinyl [K7(100)]	P54071	NA	NA	NA	NA	18,48324	18,33425	NA	NA	NA	NA	NA	NA
MIAEAIPELKASIK	1xDimethyl [K14]; 1xDimethyl [N-Term]; 1xSuccinyl [K10(100)]	P08249	NA	NA	NA	NA	14,44359	16,16065	NA	NA	NA	NA	NA	NA
NLGIGKITPFEEK	1xDimethyl [K13]; 1xDimethyl [N-Term]; 1xSuccinyl [K6(100)]	P08249	17,23983	17,6055	18,57815	18,30478	NA	NA	16,45785	16,93658	16,92101	17,3077	18,05722	17,58872
SDGKISEQSDAK	1xDimethyl [K12]; 1xDimethyl [N-Term]; 1xSuccinyl [K4(100)]	Q03265	18,4493	18,34472	19,43602	19,24958	20,92031	21,04944	19,12064	19,02342	17,85744	17,94585	18,92363	18,69873
SFLLKDPETSQR	1xDimethyl [N-Term]; 1xSuccinyl [K5(100)]	Q9CZN7	17,61255	17,5493	18,78537	18,5913	19,71905	19,83917	NA	NA	NA	NA	NA	NA
SGKPGGDVR	1xDimethyl [N-Term]; 1xSuccinyl [K3(100)]	Q9D0K2	NA	NA	NA	NA	19,08369	16,61211	NA	NA	NA	NA	NA	NA
SQGKVLQATVVAVGSGGK	1xDimethyl [K18]; 1xDimethyl [N-Term]; 1xSuccinyl [K4(100)]	Q9JI95	NA	NA	NA	NA	NA	NA	17,06	16,51771	17,50732	16,28539	17,06495	17,06173
TIAPALVSKK	1xDimethyl [K]; 1xDimethyl [N-Term]; 1xSuccinyl [K]	P17182	NA	NA	NA	NA	18,42803	18,79912	NA	NA	NA	NA	NA	NA
TVDPKTGK	1xDimethyl:2H(4) [N-Term]; 1xDimethyl:2H(4) [K8]; 1xSuccinyl [K5(100)]	Q9CZN7	NA	NA	NA	NA	12,6574	20,03871	NA	NA	NA	NA	NA	NA
VIPSPFKYADVVTTTTHK	1xDimethyl [K18]; 1xDimethyl [N-Term]; 1xSuccinyl [K7(100)]	Q9CZN7	17,45842	16,49663	18,00097	15,92055	19,02929	16,68923	NA	NA	NA	NA	NA	NA
VLDSGAPIKIPVGPETLGR	1xDimethyl [N-Term]; 1xSuccinyl [K9(100)]	P56480	17,72145	16,95358	17,41915	15,22011	20,42199	20,45106	17,47046	14,57926	15,2602	14,6559	18,34084	13,51708
VLELVSITANKNTCPGDR	1xCarbamidomethyl [C14]; 1xDimethyl:2H(4) [N-Term]; 1xSuccinyl [K11(100)]	Q9CZN7	NA	NA	NA	NA	15,90382	17,92792	NA	NA	NA	NA	NA	NA
VNVPVIGGHAGKTIIPLISQCTPK	1xCarbamidomethyl [C21]; 1xDimethyl [K24]; 1xDimethyl [N-Term]; 1xSuccinyl [K12(100)]	P08249	NA	NA	NA	NA	18,29474	18,49914	NA	NA	NA	NA	NA	NA

Table S2. Results of the statistical analysis aiming to determine RANKL induced changes on protein succinylation.

Control_1	RANKL_1	Control_2	RANKL_2	Control_3	RANKL_3	Control_4	RANKL_4	Control_5	RANKL_5	Control_6	RANKL_6	p.values	p.values.ad	Accession	Sequence	Modificati	Protein Se	Succinylation Site	Gene	Label	ratio	RatioLog	Significanc	Regulation
16,93791	16,47579	18,06801	15,01454									0,205312	0,38496	D3Z7P3	FITALKSTGL	1xDimethy	MMRLRGS	169	Gls	Gls_169	0,310328	-1,68813	not_signific	not_regualted
						17,2573	13,76659	18,81644	15,16713	15,75998	15,11015	0,079436	0,297886	P08249	ANVKGYLG	1xCarbami	MLSALARP	78	Mdh2	Mdh2_78	0,127777	-2,96829	Significant	Downregulated
17,23983	17,6055	18,57815	18,30478			16,45785	16,93658	16,92101	17,3077	18,05722	17,58872	0,832735	0,961916	P08249	NLGIGKITP	1xDimethy	MLSALARP	307	Mdh2	Mdh2_307	0,975828	-0,0353	not_signific	not_regualted
16,87558	16,89666	17,95359	18,55035	19,34497	20,40042	17,40612	14,50683	14,71989	14,01378	17,44391	14,49071	0,525838	0,876397	P38647	AQFEGIVTD	1xDimethy	MISASRAA	360	Hspa9	Hspa9_360	1,376188	0,460677	not_signific	not_regualted
				15,64505	20,44702	19,75541	18,58995	18,87179	18,14027	19,73062	19,35954	0,591243	0,886865	P51881	DFLAGGVA	1xDimethy	MTDAAVSF	23	Slc25a5	Slc25a5_2	1,218552	0,285168	not_signific	not_regualted
17,72145	16,95358	17,41915	15,22011	20,42199	20,45106	17,47046	14,57926	15,2602	14,6559	18,34084	13,51708	0,161562	0,375645	P56480	VLDSGAPIK	1xDimethy	MLSLVGRV	133	Atp5f1b	Atp5f1b_1	0,706896	-0,50043	not_signific	not_regualted
16,81741	17,1779	17,92034	17,45141									0,938103	0,961916	P56480	GQKVLDSG	1xDimethy	MLSLVGRV	124	Atp5f1b	Atp5f1b_1	0,900826	-0,15068	not_signific	not_regualted
18,4493	18,34472	19,43602	19,24958	20,92031	21,04944	19,12064	19,02342	17,85744	17,94585	18,92363	18,69873	0,916582	0,961916	Q03265	SDGKISEQS	1xDimethy	MLSVRVAA	531	Atp5f1a	Atp5f1a_5	0,99561	-0,00635	not_signific	not_regualted
18,74808	18,51845	19,65044	19,14292	20,31967	17,12203	18,3971	14,74575	17,6744	14,46401	18,36898	14,4103	0,037644	0,205824	Q9CZN7	LQDFKSFLL	1xDimethy	MVSFSLLRT	469	Shmt2	Shmt2_46	0,337354	-1,56766	Significant	Downregulated
17,45842	16,49663	18,00097	15,92055	19,02929	16,68923							0,041165	0,205824	Q9CZN7	VIPSPFKYA	1xDimethy	MVSFSLLRT	269	Shmt2	Shmt2_26	0,266162	-1,90962	Significant	Downregulated
17,61255	17,5493	18,78537	18,5913	19,71905	19,83917							0,961916	0,961916	Q9CZN7	SFLLKDPET	1xDimethy	MVSFSLLRT	474	Shmt2	Shmt2_47	1,006251	0,008991	not_signific	not_regualted
16,15054	17,10366	15,38256	17,57019	15,5349	20,87515	16,11394	17,27022	14,43834	17,88855	16,31943	17,66756	0,006566	0,098491	Q9CZU6	GMKGLVYE	1xDimethy	MALLTAAT	76	Cs	Cs_76	8,488261	3,085469	Significant	Upregulated
16,44119	17,06161	15,11181	17,26002	15,84152	18,37742	15,61939	15,91522	14,75482	16,27171	15,65919	14,22471	0,175301	0,375645	Q9CZU6	GMKGLVYE	1xOxidatio	MALLTAAT	76	Cs	Cs_76	2,539123	1,344331	not_signific	not_regualted
16,94037	17,09634	18,34704	18,29486									0,960433	0,961916	Q9CZU6	IVPNILLEQ	1xDimethy	MALLTAAT	0	Cs	Cs_0	1,005474	0,007875	not_signific	not_regualted
						17,06	16,51771	17,50732	16,28539	17,06495	17,06173	0,10937	0,32811	Q9JI95	SQGKVLQA	1xDimethy	MAGQAFRK	40	Hspe1	Hspe1_40	0,674922	-0,56721	not_signific	not_regualted
